# Hexamethylene amiloride binds the SARS‐CoV‐2 envelope protein at the protein–lipid interface

**DOI:** 10.1002/pro.4755

**Published:** 2023-10-01

**Authors:** Noah H. Somberg, João Medeiros‐Silva, Hyunil Jo, Jun Wang, William F. DeGrado, Mei Hong

**Affiliations:** ^1^ Department of Chemistry Massachusetts Institute of Technology Cambridge Massachusetts USA; ^2^ Department of Pharmaceutical Chemistry University of California San Francisco San Francisco California USA; ^3^ Department of Medicinal Chemistry, Ernest Mario School of Pharmacy, Rutgers the State University of New Jersey Piscataway New Jersey USA

**Keywords:** COVID‐19, drug binding, SARS‐CoV‐2 envelope, solid‐state NMR, viroporin

## Abstract

The SARS‐CoV‐2 envelope (E) protein forms a five‐helix bundle in lipid bilayers whose cation‐conducting activity is associated with the inflammatory response and respiratory distress symptoms of COVID‐19. E channel activity is inhibited by the drug 5‐(N,N‐hexamethylene) amiloride (HMA). However, the binding site of HMA in E has not been determined. Here we use solid‐state NMR to measure distances between HMA and the E transmembrane domain (ETM) in lipid bilayers. ^13^C, ^15^N‐labeled HMA is combined with fluorinated or ^13^C‐labeled ETM. Conversely, fluorinated HMA is combined with ^13^C, ^15^N‐labeled ETM. These orthogonal isotopic labeling patterns allow us to conduct dipolar recoupling NMR experiments to determine the HMA binding stoichiometry to ETM as well as HMA‐protein distances. We find that HMA binds ETM with a stoichiometry of one drug per pentamer. Unexpectedly, the bound HMA is not centrally located within the channel pore, but lies on the lipid‐facing surface in the middle of the TM domain. This result suggests that HMA may inhibit the E channel activity by interfering with the gating function of an aromatic network. These distance data are obtained under much lower drug concentrations than in previous chemical shift perturbation data, which showed the largest perturbation for N‐terminal residues. This difference suggests that HMA has higher affinity for the protein–lipid interface than the channel pore. These results give insight into the inhibition mechanism of HMA for SARS‐CoV‐2 E.

## 1 INTRODUCTION

**FIGURE 1:**
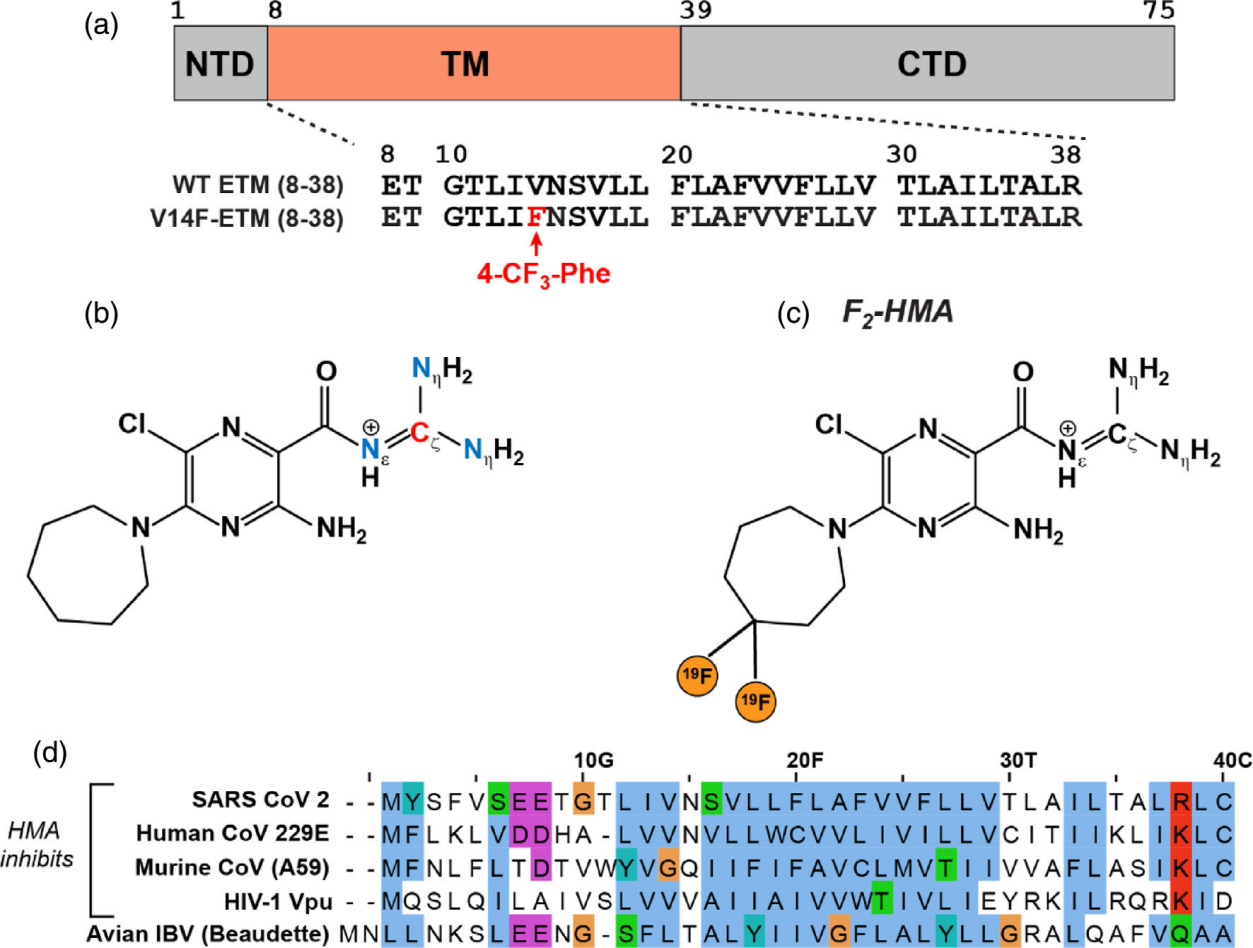
SARS‐CoV‐2 E amino acid sequences, HMA structure, and isotopic labeling schemes used in this work. (a) Sequence diagram of the SARS‐CoV‐2 E protein and the amino acid sequence of the TM domain. Wild‐type (WT) ETM is recombinantly expressed with a variety of isotopic labeling patterns. V14F‐ETM contains a synthetically incorporated CF_3_‐Phe14 mutation. (b) Structure of guanidinium ^13^C, ^15^N‐labeled HMA (CN‐HMA). (c) Structure of 4,4‐difluoro‐HMA (F_2_‐HMA). (d) Comparison of the TM amino acid sequences of HMA‐inhibited viroporins and the noninhibited IBV E protein. Sequences were aligned and colored with the ClustalW service in JalView v2.11 (Larkin et al., 2007; Waterhouse et al., 2009).

From 2020 to 2023, the COVID‐19 pandemic has caused 700 million confirmed infections and nearly 7 million confirmed deaths (WHO, 2023). Still only a handful of treatments are available, including Paxlovid, which targets the main protease of the SARS‐CoV‐2 virus, and Remdesivir and Molnupiravir, which target the RNA polymerase (Li et al., 2023). The search for new antiviral drugs continues to be an important goal as COVID‐19 becomes endemic. One potential target of antiviral drugs is the envelope (E) protein (Nieto‐Torres et al., 2014; Nieto‐Torres et al., 2015; Xia et al., 2021), one of the three structural membrane proteins encoded by the viral genome. Of these, the E protein is the smallest and the least understood. The 75‐residue E consists of a transmembrane (TM) domain flanked by a short N‐terminal ectodomain and a C‐terminal domain (Figure 1a). E assembles into a homo‐pentamer (Li et al., 2014; Parthasarathy et al., 2008; Parthasarathy et al., 2012; Somberg et al., 2022; Torres et al., 2005) that is mainly localized to the endoplasmic reticulum‐Golgi intermediate compartment (ERGIC) of the cell (Lopez et al., 2006; Nieto‐Torres et al., 2011). Here E acts as a cation channel (Liao et al., 2004; Wilson et al., 2004) that disrupts cell homeostasis and triggers the inflammatory responses of the cell to viral infection (Nieto‐Torres et al., 2015). The E protein also senses membrane curvature (Mehregan et al., 2022), participates in virus assembly and egress (Nieto‐Torres et al., 2014), and interacts with other viral and host proteins through its C‐terminal domain (Chai et al., 2021; Jimenez‐Guardeno et al., 2014; Zheng et al., 2021).

Amiloride is a small‐molecule drug that blocks the epithelial sodium channel (Kleyman & Cragoe, 1988). The molecule is composed of a pyrazine ring substituted with an acyl guanidinium group. Since its initial synthesis (Cragoe et al., 1967), more than a thousand amiloride analogs with varying substitutions of the pyrazine ring have been produced to inhibit a wide range of membrane transport processes, enzymes, and DNA and RNA synthesis (Kleyman & Cragoe, 1988). One such derivative, 5‐(N,N‐hexamethylene) amiloride (HMA) (Figure 1b,c), inhibits the ion channel activity of the human immunodeficiency virus 1 (HIV‐1) viral protein U (Vpu) (Figure 1d) (Ewart et al., 2002; Ewart et al., 2004). After the first SARS outbreak in 2002, HMA has been studied as an inhibitor of SARS‐CoV E, which has the same TM amino acid sequence as SARS‐CoV‐2 E. Channel current measurements in planar bilayers showed that HMA blocks the E current while virus plaque assays indicate that the compound inhibits the replication of the virus (Pervushin et al., 2009; Wilson et al., 2006; Xia et al., 2021). The IC_50_ of HMA for SARS‐CoV E is about 10 μM (Pervushin et al., 2009; Xia et al., 2021).

To determine the HMA binding site in the SARS‐CoV E protein, NMR chemical shift perturbations (CSP) have been measured on various E constructs that are reconstituted in membrane‐mimetic solvents (Li et al., 2014; Park et al., 2021; Pervushin et al., 2009; Toft‐Bertelsen et al., 2021). Most of these studies showed that the CSPs are concentrated in the N‐terminal region of the TM domain between residues 8 and 15. Solution NMR experiments were conducted on micelle‐bound E proteins that either contain only the TM domain or include both the TM domain and the cytoplasmic region. Most CSPs were measured under large excess of HMA, with a protein monomer to drug molar ratio (P:D) of 1:10. Given the pentameric nature of the E assembly in the lipid membrane, this corresponds to a pentamer to drug ratio of 1:50. Recently, the structure of ETM at neutral pH in lipid bilayers was studied using solid‐state NMR (ssNMR). The data show that HMA binding caused the largest CSPs for N‐terminal residues of the TM domain (Mandala et al., 2020). These CSPs were observed at a P:D ratio of 1:4. When the drug concentration decreased to a P:D ratio of 1:1, the CSPs became negligible. To date, no study has directly measured the distance contacts between HMA and protein residues, or the contact between HMA and lipids.

To elucidate the inhibition mechanism of HMA to the E protein, here we investigate the binding site and binding stoichiometry of HMA in membrane‐bound ETM using ssNMR. For this purpose, we prepared a panel of membrane samples that contain orthogonal isotopically labeled protein and drug. These include ^13^C, ^15^N‐labeled ETM combined with fluorinated HMA, and fluorinated protein combined with ^13^C‐labeled HMA. HMA‐containing proteoliposomes were examined at pH 7.5, which corresponds to a poorly hydrated “closed” state of the channel, and at pH 4.5, which corresponds to a well hydrated “open” state of the channel (Medeiros‐Silva et al., 2022). Using ^13^C–^19^F, ^1^H–^19^F, and ^13^C–^15^N rotational‐echo double resonance (REDOR) NMR experiments that measure internuclear distances, we determined the binding stoichiometry of HMA to ETM and obtained distance restraints between the drug and the protein. These distance restraints involve both the guanidinium polar head and the hydrophobic hexamethylene ring of HMA. Unexpectedly, these distance data show that HMA contacts residues in the middle of the TM domain on the lipid‐facing surface of the protein, rather than occupying the N‐terminal pore of the channel. This finding has important implications for the mechanism of inhibition of ETM by HMA.

## 2 RESULTS

### 2.1 Conformation and dynamics of membrane‐bound HMA

ETM spans residues 8–38 of the full‐length E protein (Figure 1a) and is highly hydrophobic, containing 16 Leu, Val and Ile residues and three Phe residues. We expressed recombinant wild‐type ETM containing the desired ^13^C and ^15^N labels, and additionally synthesized an ETM peptide in which V14 is replaced by 4‐CF_3_‐Phe. This V14F‐ETM construct allows us to use the CF_3_ group to measure HMA contact with the N‐terminal region of the protein with high spectral sensitivity. HMA is an elongated and approximately planar molecule that connects the polar guanidinium and the hydrophobic hexamethyelene ring (also called azepane) by a chlorine and amine‐substituted pyrazine. We produced two isotopically labeled HMA compounds in this work: a guanidinium ^13^C, ^15^N‐labeled HMA (Figure 1b) and a ring‐difluorinated F_2_‐HMA (Figure 1c). These samples allow us to probe the binding sites of the drug in ETM using a variety of intermolecular distance experiments.

**FIGURE 2:**
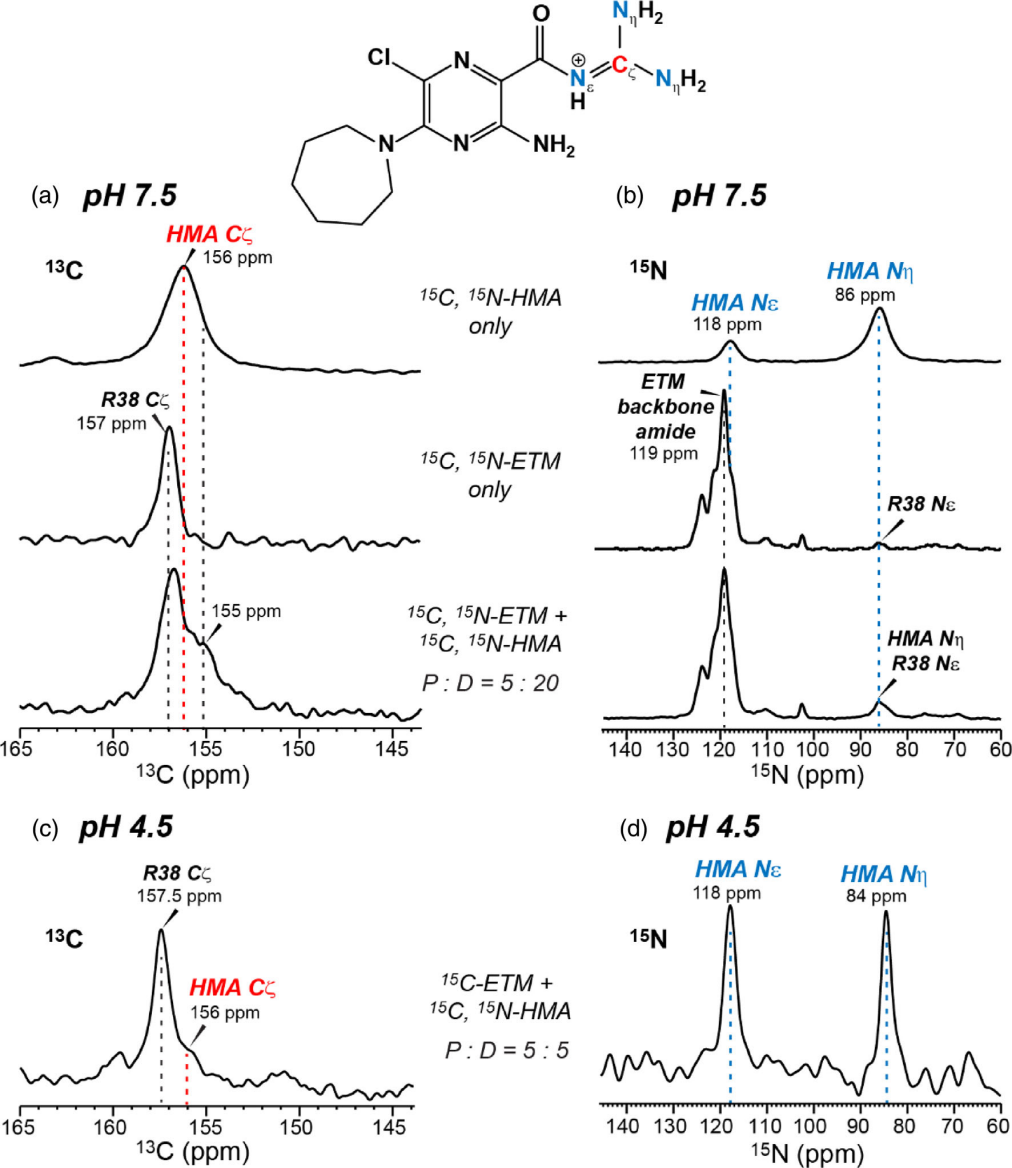
^13^C and ^15^N CP spectra of ^13^C, ^15^N‐labeled HMA and ETM separately and in combination, reconstituted in DMPC/DMPG membranes. The pH 7.5 proteoliposomes correspond to the less hydrated “closed” state of ETM whereas the pH 4.5 proteoliposomes represent the more hydrated “open” state. (a) ^13^C CP spectra of membrane‐bound ^13^C, ^15^N‐labeled HMA alone (top row), ^13^C, ^15^N‐labeled ETM alone (middle row), and ^13^C, ^15^N‐labeled ETM and HMA together (bottom row). The acyl guanidium Cζ signal of HMA partially overlaps with the protein R38 Cζ signal. All three samples were at pH 7.5 and were measured in the gel‐phase membrane around 260 K. The protein/drug molar ratio (P:D) is 5:20 in the complex. (b) ^15^N spectra of the same samples as in (a). The HMA Nε signal partially overlaps with the protein amide H^N^ signal, whereas the HMA Nη peak at 86 ppm overlaps with the R38 Nε signal. (c) ^13^C CP spectrum of ^13^C‐labeled ETM and ^13^C, ^15^N‐labeled HMA at pH 4.5. (d) ^15^N CP spectrum of the same sample as in (c). Since the protein is not ^15^N labeled, both peaks in this spectrum result from HMA.

We first characterized the conformation and dynamics of HMA using 1D ^13^C, ^15^N, and ^19^F experiments. These experiments probe the properties of the drug and the protein separately and in combination, all in the DMPC/DMPG lipid membrane. In the absence of the protein, the HMA guanidinium exhibits a Cζ chemical shift of 156 ppm, an Nε chemical shifts of 118 ppm, and an Nη chemical shift of 86 ppm (Figure 2). In the gel phase of the membrane at 260 K, the ^13^C and ^15^N linewidths are 2.3 ppm and 5 ppm, respectively, indicating that the polar end of HMA adopts a distribution of conformations. In comparison, membrane‐bound ETM shows an R38 Cζ chemical shift of 157 ppm and a bulk amide ^15^N chemical shift of 119 ppm (Figure 2b). Thus, the HMA Cζ chemical shift is 1.0–1.5 ppm smaller than the R38 Cζ chemical shift, whereas the HMA Nε chemical shift is 1.0 ppm smaller than the protein amide ^15^N chemical shift. Given the similar chemical shifts of the HMA guanidinium and R38 of the protein, we chose different isotopic labeling schemes between the protein and the drug to measure protein–drug contacts unambiguously.

**FIGURE 3:**
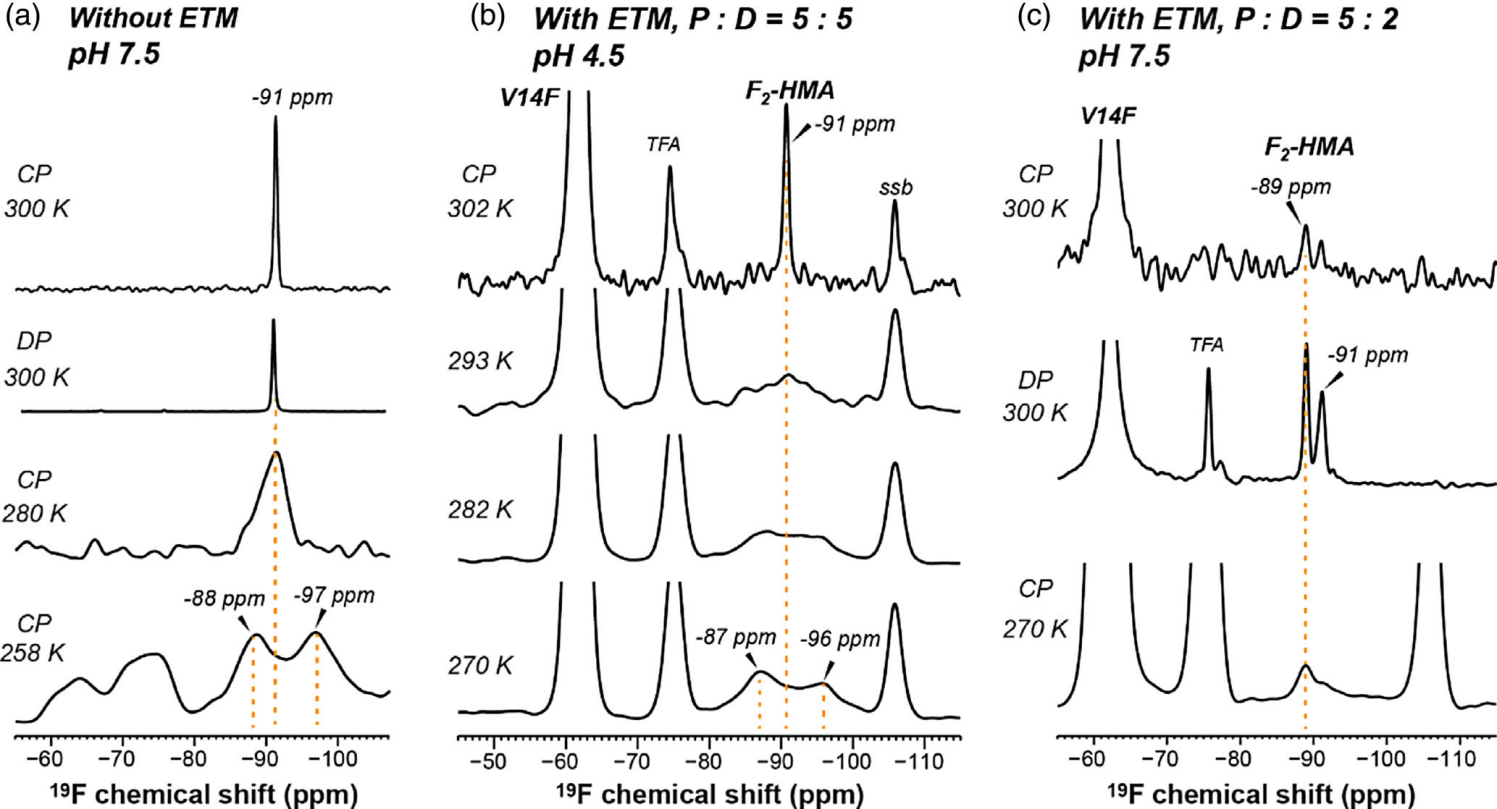
^19^F NMR spectra of DMPC/DMPG membrane‐bound F_2_‐HMA. (a) ^19^F CP and DP spectra of membrane‐bound F_2_‐HMA in the absence of the protein. A single ^19^F is observed at high temperature in the liquid‐crystalline phase of the membrane. Cooling the sample to the gel phase broadened the peaks and caused peak doubling at 258 K. (b) ^19^F CP spectra of F_2_‐HMA in complex with V14F‐CF_3_‐ETM at a P:D ratio of 5:5 and pH 4.5. At 302 K, HMA exhibits a single ^19^F peak at −91 ppm. From 293 K to 270 K, the ^19^F signal broadens and splits into two peaks. The line broadening occurs at higher temperature than the peptide‐free sample. The strong −62 ppm peak is from the peptide CF_3_, the −75 ppm peak is from residual TFA, and the −106 ppm peak is due to a spinning sideband (ssb). (c) ^19^F spectra of F_2_‐HMA in complex with V14‐CF_3_‐ETM at a P:D ratio of 5:2 and pH 7.5. Two ^19^F signals are resolved at high temperature, indicating two conformations of the hexamethylene ring under this condition.

While ^13^C, ^15^N‐labeled HMA provides information about the polar end of the molecule, ring‐fluorinated HMA probes the structural dynamics of the hydrophobic end. Figure 3 shows the ^19^F NMR spectra of membrane‐bound F_2_‐HMA as a function of temperature and in the absence or presence of the protein. In the absence of ETM, HMA exhibits a narrow ^19^F peak at −91 ppm at high temperature (Figure 3a,c). This peak is observed in both CP and direct‐polarization (DP) spectra, indicating that the hexamethylene ring is anisotropically mobile in the liquid‐crystalline phase of the membrane. With decreasing temperature, the ^19^F lineshape broadens severely: at 280 K, the linewidth increased to 4.4 ppm, whereas at 258 K, two peaks are resolved at −87 ppm and −96 ppm. This temperature‐dependent line broadening indicates that the hexamethylene ring undergoes fast exchange among many conformations at high temperature, which are frozen in the gel‐phase membrane. The peak doubling at low temperature suggests that the two C—F bonds have different conformational environments, which become resolved when the ring is immobilized.

When ETM is present in the membrane, the HMA ^19^F chemical shifts show interesting lineshape changes. At a P:D ratio of 5:5 at low pH, HMA exhibits the same ^19^F chemical shift of −91 ppm at high temperature and the same peak doubling at low temperature as the protein‐free sample (Figure 3b). However, the ^19^F line broadening shifts to higher temperatures compared to the protein‐free sample. At 270 K, the ^19^F spectrum already exhibits peak doubling, indicating that the presence of the protein immobilizes the drug. When the drug concentration decreased to a P:D ratio of 5:2 at pH 7.5, we observed two ^19^F peaks at −89 ppm and −91 ppm even at high temperature (Figure 3c). Since the −91 ppm peak corresponds to the lipid‐interacting HMA, we attribute the −89 ppm peak to protein‐interacting HMA. The drug‐detected ^13^C–^19^F REDOR data below (*vide infra*) indicates that one HMA binds five ETM helices. Thus, the P:D ratio of 5:5 means that the sample has four‐fold excess drug whereas the 5:2 sample has one‐fold excess drug. The fact that the 5:5 sample shows a predominant ^19^F peak with the same chemical shift as the lipid‐bound HMA can thus be attributed to the larger excess of the drug in this sample. These data, taken together, indicate that protein‐bound HMA has a small ^19^F chemical shift difference of ~2 ppm from the lipid‐bound HMA, and more drug interacts with the lipids in the 5:5 sample than in the 5:2 sample. Below, the distance measurements directly verify this interpretation.

### 2.2 HMA binding to ETM detected from chemical shift perturbation and distance measurements

Our recent solid‐state NMR study of HMA interaction with ETM used chemical shift perturbation of the protein as a qualitative indicator of the drug binding site (Mandala et al., 2020). These chemical shifts were measured at a protein monomer to drug molar ratio of 1:4 (Mandala et al., 2020). Given the pentameric nature of the channel, this ratio corresponds to 20 equivalents of HMA per pentamer. The spectra showed that residues T9, G10, and T11 near the N‐terminus have the largest CSPs of 0.35–0.70 ppm per residue. S16 also exhibits a non‐negligible CSP of 0.23 ppm. Near the C‐terminus, residues A36 and L37 show moderate CSPs of 0.11 ppm. These results suggest that under those sample conditions, HMA interacts with the N‐terminal region of the channel pore. However, CSPs can be induced allosterically. Moreover, since HMA readily partitions into the lipid membrane (Figures 2 and 3a), in principle it can bind ETM from the membrane side. Therefore, to definitively locate the drug binding site in the protein, intermolecular distance measurements are required. In this study, we employ REDOR (Gullion & Schaefer, 1989) as the main technique to measure distances between HMA and ETM.

**FIGURE 4:**
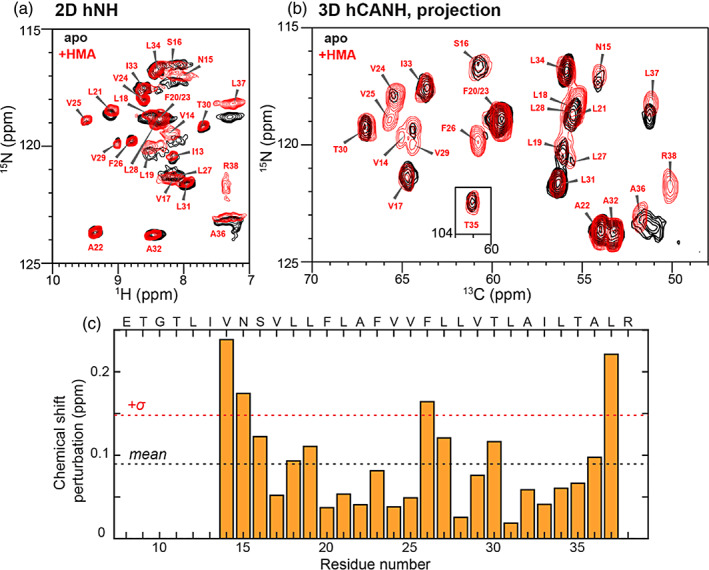
Chemical shift perturbation by F_2_‐HMA. The spectra of F_2_‐HMA bound ETM at pH 4.5 in DMPC/DMPG membrane (red contours) are compared with the spectra of drug‐free protein at pH 4.5 with 20 mM Ca^2+^ in ERGIC‐mimetic membrane (black contours). The HMA‐bound protein has a P:D ratio of 5:5. Both spectra were measured on the 600 MHz NMR under 55 kHz MAS at a sample temperature of 276 K. (a) 2D hNH of the apo and F_2_‐HMA bound ETM. (b) 2D projection of the 3D hCANH spectra of the apo and F_2_‐HMA bound ETM. (c) Combined ^1^H, ^13^C, and ^15^N chemical shift perturbations by HMA. Black dashed line denotes the average CSP while the red dashed line indicates the boundary for one standard deviation from the mean.

To verify whether the distance measurement and CSP measurement reflect the same state, we measured the HMA‐induced CSPs in one of the samples used for distance measurements. Using the pH 4.5 sample with a P:D of 5:5 (Table S1), we measured ^1^H‐detected 2D hNH and 3D hCANH spectra (Figure 4a,b) and compared them with the spectra of drug‐free ETM at low pH. We observed relatively large CSPs for V14, N15, F26, and L37. R38 was immobilized in the drug‐bound sample but was too dynamic to be detected in the apo sample. The combined ^1^H, ^13^C and ^15^N CSPs per residue (Figure 4c) are the largest for the N‐ and C‐terminal residues. This observation is in good agreement with the CSP trend reported before, even though the previous studies used larger excess of HMA and both detergent micelles and lipid bilayers (Li et al., 2014; Park et al., 2021; Pervushin et al., 2009; Toft‐Bertelsen et al., 2021). Interestingly, in addition to the terminal residues, we also observed significant CSPs at F26 and L27 in the middle of the TM peptide. Moreover, V24, V25, F26, and V29 peaks became sharper in the presence of HMA than in its absence, indicating that residues in the middle of the TM domain become more ordered upon drug binding.

**FIGURE 5:**
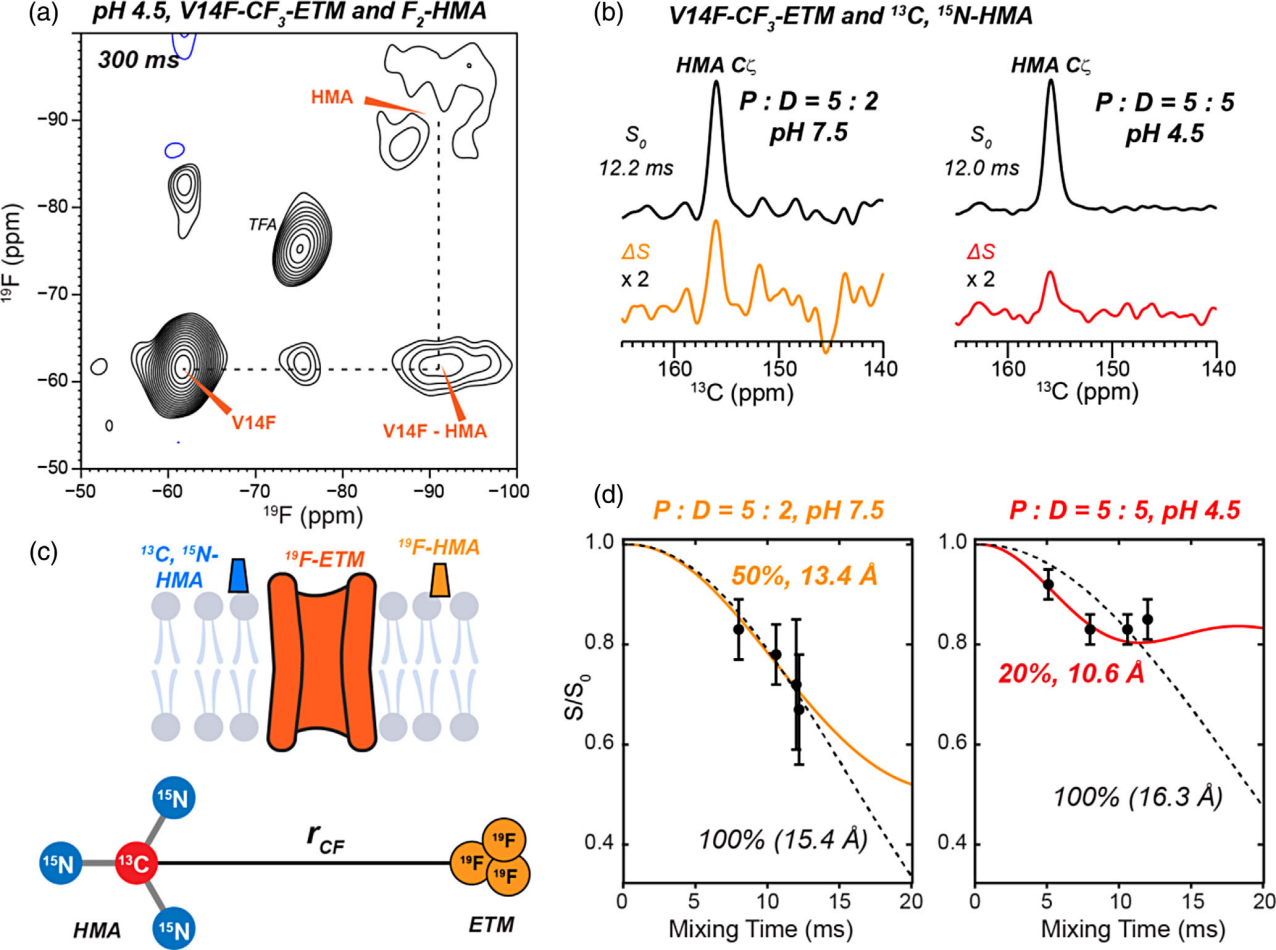
Distance contacts between HMA and ETM from 2D ^19^F–^19^F correlation and ^13^C–^19^F REDOR experiments. (a) 2D ^19^F–^19^F correlation spectrum with 300 ms mixing. V14F‐CF_3_‐labeled ETM is complexed with F_2_‐HMA at pH 4.5 at a P:D ratio of 5:5. A cross peak between CF_3_ and HMA cross peak is observed, indicating that the HMA fluorines are within ~2 nm of the peptide CF_3_. (b) Representative ^13^C–^19^F REDOR *S*_0_ and ΔS spectra of ^13^C, ^15^N‐labeled HMA and V14F‐CF_3_‐labeled ETM at pH 7.5 (left) and pH 4.5 (right). (c) Schematic of ^13^C, ^15^N‐labeled HMA and ^19^F‐labeled ETM for distance measurements. HMA is sketched outside the membrane to avoid biasing its position in the membrane or in the channel pore prior to experimental analysis. The two‐spin model used for simulating ^13^C–^19^F REDOR dephasing is shown. The three CF_3_ fluorines are modeled as a single pseudo‐fluorine whose distance (*r*_CF_) to the guanidinium Cζ is obtained from fitting the data. (d) Measured and best‐fit ^13^C–^19^F REDOR dephasing, For the P:D = 5:2 sample at pH 7.5, 50% of the HMA guanidinium ^13^C is dephased by the protein ^19^F. For the P:D = 5:5 sample at pH 4.5, 20% of the HMA ^13^C signal is dephased by the protein ^19^F. Thus, these data indicate a binding stoichiometry of one HMA per ETM pentamer.

With the chemical shift perturbations largely reproducing the previous data, we next investigated if HMA is within nanometer contact of ETM using a 2D ^19^F spin diffusion NMR experiment. ^19^F spin diffusion is sensitive to internuclear distances of ~2 nm (Roos et al., 2018). V14F‐CF_3_‐labeled ETM is complexed with F_2_‐HMA and a 2D ^19^F spin diffusion spectrum was measured using a long mixing time of 300 ms to maximally observe potential contact between HMA and residue 14 (Figure 5a). The CF_3_ group of Phe14 resonates at −62 ppm, which is well resolved from the F_2_‐HMA chemical shift of −91 ppm. The 2D spectrum shows a clear cross‐peak from CF_3_ to HMA fluorines, indicating that HMA is indeed within ~2 nm of residue 14. The 2D spectrum is asymmetric, with no cross‐peak from the HMA fluorines to the CF_3_. This asymmetry has been observed before (Roos et al., 2018) and may result from dipolar truncation between the two geminal fluorines, which would slow down magnetization transfer to the CF_3_ group.

Since HMA has affinity for both the protein and the lipids, we sought to determine the binding stoichiometry of the drug to ETM pentamers. We prepared membrane samples with P:D ratios of 5:2 and 5:5 while keeping the protein/lipid ratio at 5:50. For the 5:5 samples, if one HMA binds each pentamer whereas the other four equivalents of drug bind lipids, then only 20% of the drug should experience substantial dipolar couplings to the protein. In a REDOR experiment that detects the drug signals, if all drug is in close contact with the protein, then the REDOR intensity should decay to an *S*/*S*_0_ value of 0. But if only 20% of the drug is in close contact with the protein, then the dipolar dephasing should plateau to an *S*/*S*_0_ value of 0.80. For the same reason, in a 5:2 sample, if only one of the two equivalents of the drug is in close contact with the protein, then the drug‐detected REDOR intensities should decay to 0.50.

We measured the ^13^C–^19^F REDOR spectra of guanidinium ^13^C‐labeled HMA mixed with V14F‐CF_3_‐ETM to determine the binding stoichiometry. Both 5:2 and 5:5 samples exhibit substantial REDOR dephasing of the drug by the protein (Figure 5b), consistent with the 2D ^19^F–^19^F spin diffusion result that HMA is in molecular contact with the protein. The REDOR *S*/*S*_0_ values decayed to 0.65 for the 5:2 sample at the longest mixing time measured and to 0.85 for the 5:5 sample. These results indicate that HMA binds ETM with a stoichiometry of approximately one drug for five helices. This was observed at both high and low pH, indicating that the closed and open states of the channel do not affect drug binding appreciably. Since ETM assembles into five‐helix bundles, the best interpretation of this result is that one drug binds each pentamer.

With the binding stoichiometry known, we next quantified the drug–protein distances (*d*_CF_) by fitting the measured ^13^C–^19^F REDOR dephasing using a two‐spin model (Figure 5c, Figure S3a). The model consists of the guanidinium Cζ and a pseudo‐fluorine that is equivalent to the three fluorines in the rotating CF_3_ group (Elkins et al., 2017). This pseudo‐fluorine has 3^1/2^ times the dipolar coupling of a single fluorine to the ^13^C. Taking into account the intensity scaling of 0.50 for the binding stoichiometry of the 5:2 sample and 0.20 for the binding stoichiometry of the 5:5 sample, the measured ^13^C–^19^F REDOR dephasing is best fit to a distance of 13.4 Å for the 5:2 complex and 10.6 Å for the 5:5 complex between HMA Cζ and Phe14 CF_3_ (Figure 5d).

**FIGURE 6:**
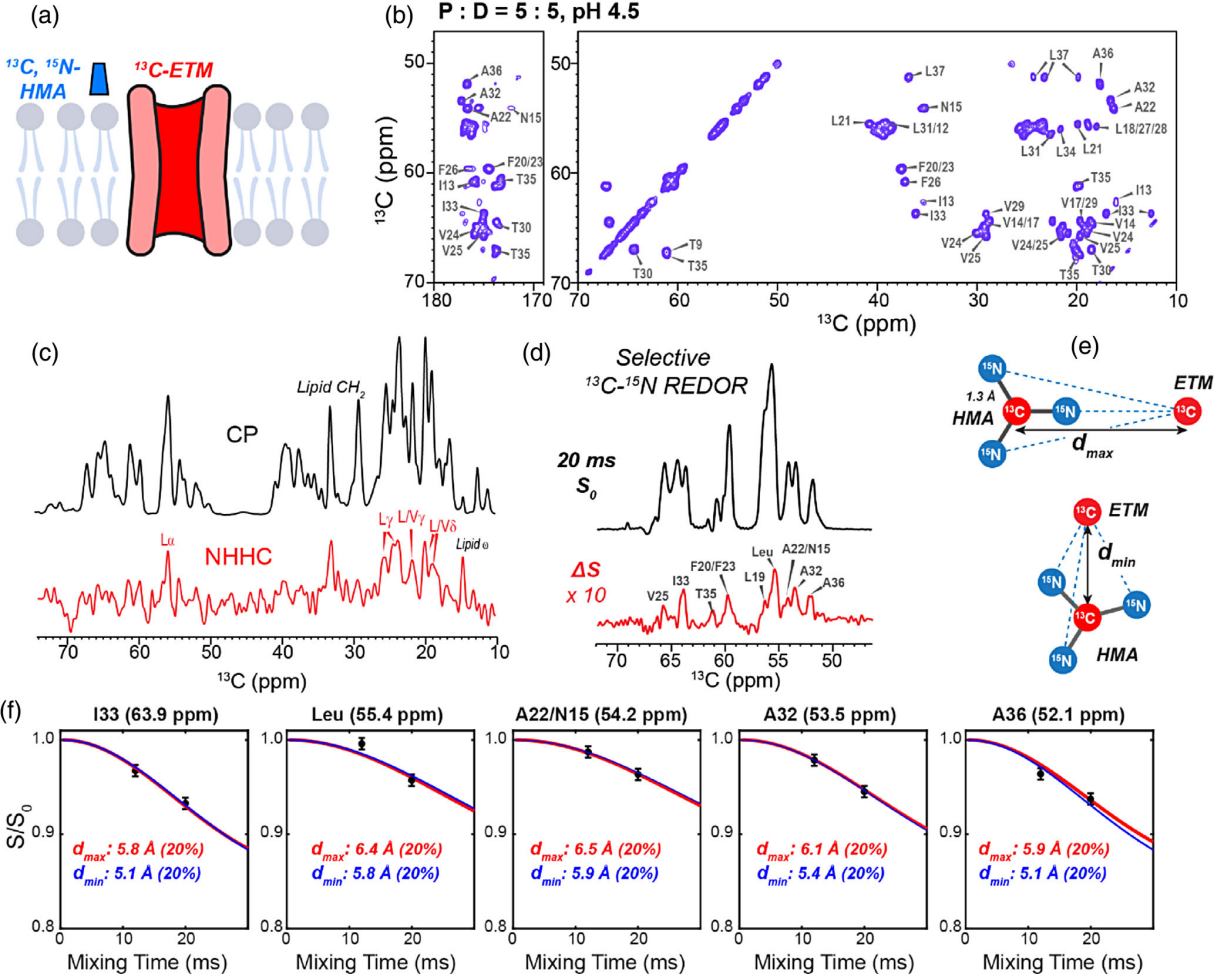
Measurement of ^13^C–^15^N distances between HMA and ETM. (a) Schematic of ^13^C‐labeled ETM and ^15^N‐labeled HMA for distance measurements. (b) 2D ^13^C–^13^C correlation spectrum of ^13^C‐labeled ETM with bound HMA (P:D = 5:5) measured with a CORD mixing time of 23 ms. Resonance assignments are indicated. (c) ^13^C CP spectrum and 1D NHHC spectrum of HMA‐bound ETM. The NHHC spectrum was measured with ^1^H mixing times of 0.5 ms and 1 ms. Protein and lipid ^13^C signals that are transferred from the HMA ^15^N are assigned. (d) Selective ^13^Cα‐^15^N REDOR spectra of HMA‐bound ETM. The difference (ΔS) signals indicate HMA‐proximal protein ^13^C sites. (e) Geometric models for simulating ^13^C–^15^N REDOR dephasing. The maximum distance *d*_max_ between a protein ^13^C and HMA nitrogens for an observed dephasing is achieved by a lateral approach of the protein ^13^C to the guanidinium, whereas the minimum distance *d*_min_ is achieved by a vertical approach of the protein ^13^C to the guanidinium. (f) Best‐fit ^13^C–^15^N REDOR simulations for representative measured dipolar dephasing. Best‐fit curves for maximum distances (red curves) and minimum distances (blue curves) are overlaid with the experimental data. The simulated REDOR intensities are scaled by 20% to reflect the case of one HMA lying the closest to one of the five ETM helices.

These contacts between Phe14 and HMA can in principle result from HMA bound either inside the N‐terminal pore or on the lipid‐facing surface of the protein. The former would resemble amantadine binding to the N‐terminal pore of influenza AM2 to occlude the proton‐conducting pathway (Cady et al., 2010; Stouffer et al., 2008). Because the binding stoichiometry is unchanged between acidic and neutral pH, but ETM has a more spacious pore at acidic pH (Mandala et al., 2020; Medeiros‐Silva et al., 2023), we measured additional protein–drug distances under the acidic condition. The second distance measurement was conducted on ^13^C‐labeled ETM complexed to ^13^C, ^15^N‐labeled HMA (Figure 6) at a P:D ratio of 5:5. Because HMA contains the only ^15^N spins in this mixture, we can measure protein ^13^C to drug ^15^N distances unambiguously. The maximum ^13^C–^15^N distances that can be measured are much shorter than the ^13^C–^19^F distances due to the low gyromagnetic ratio of ^15^N compared to ^19^F. This short distance reach allows us to detect specific atomic‐level interactions. The ^13^C chemical shifts of the HMA‐bound protein are well resolved, as seen in the 2D ^13^C–^13^C correlation spectrum (Figure 6b) (Mandala et al., 2020). We first measured a 1D NHHC spectrum (Lange et al., 2002) (Figure 6c) to obtain qualitative information about protein–drug contacts. The experiment transfers the ^15^N magnetization of HMA to ^13^C sites in the protein via the intervening protons. The resulting NHHC spectrum exhibits Leu Cα and sidechain methyl carbons of Leu and Val residues, confirming that HMA is in molecular contact with the protein. We also detected lipid ^13^C signals at 14 ppm and 33 ppm, consistent with the excess HMA in the sample, which partitions into the lipid membrane.

To obtain higher‐sensitivity and more quantitative distances between HMA and the protein, we turned to the frequency‐selective ^13^Cα‐^15^N REDOR experiment (Jaroniec et al., 2001). Figure 6d shows the REDOR *S*_0_ and Δ*S* spectra measured with 20 ms mixing. Several residues in the C‐terminal half of ETM, including F20/F23, A32, I33, T35, and A36, exhibit difference intensities, indicating that these residues are in close contact to the guanidinium moiety of the drug. The normalized REDOR dephasing, *S*/*S*_0_, plateaus to ~0.93. Despite the small deviation from 1.0, these *S*/*S*_0_ values are precise because of the high sensitivity of the ^13^C *S*_0_ spectra. Because the HMA‐detected ^13^C–^19^F REDOR data indicate a binding stoichiometry of one HMA for every five helices, if the one equivalent of HMA is bound centrally in the channel pore, then these protein‐detected ^13^C–^15^N REDOR intensities should decay to 0. But if HMA is bound to the surface of the pentamer, then only one or two helices may be dephased by the drug. The observed high REDOR intensities of ~0.93 in the protein‐detected REDOR spectra therefore indicate that HMA is not equidistant to all five helices, but is bound to the lipid‐facing surface of the protein, in closer contact with one of the helices than the others. Thus, we used a scaling factor of 20% to simulate the protein‐detected ^13^C–^15^N REDOR data. To simulate the ^13^C–^15^N REDOR dephasing between a protein ^13^C and three guanidinium nitrogen atoms, we employed a four‐spin system (Figure 6e, Figure S3b,c). The three ^13^C–^15^N distances are parameterized by the distance between the protein ^13^C and the guanidinium ^13^C. The measured REDOR dephasing can in principle be fit to a variety of distances, depending on the angle of approach of the protein to the guanidinium group. A lateral approach that aligns the ^13^C–^13^C vector with one of the ^13^C–^15^N bonds corresponds to the maximum possible distance, *d*_max_, whereas a vertical approach that orients the ^13^C–^13^C vector perpendicular to the plane of the three nitrogen atoms corresponds to the shortest distance, *d*_min_, for the measured dephasing. Figure 6f shows that the Cα carbons of residues 32–36 are 5.1–6.5 Å from the HMA guanidinium ^13^C, which place tight constraints on the drug location.

**FIGURE 7:**
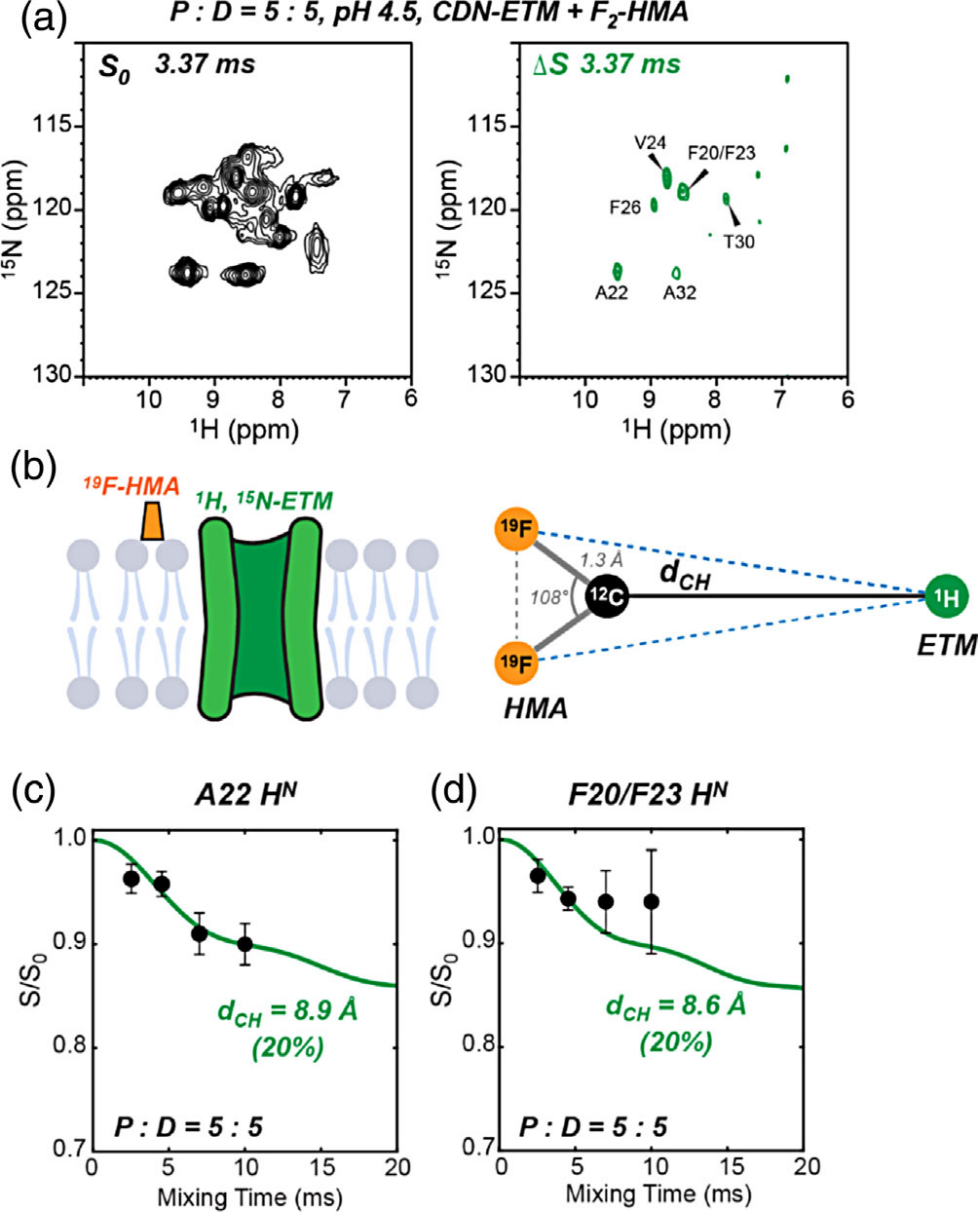
Measurement of H^N^‐F distances between ETM and HMA using the ^1^H–^19^F REDOR experiment. (a) 2D hNH‐resolved ^1^H–^19^F REDOR *S*_0_ and Δ*S* spectra of the pH 4.5 sample with P:D = 5:5. The REDOR mixing time is 3.37 ms. (b) Schematic of ^1^H, ^15^N‐labeled ETM and ^19^F‐labeled HMA for distance measurements, and three‐spin model used to simulate ^1^H–^19^F REDOR dephasing. The distance *d*_CH_ between a protein H^N^ and the fluorine‐bonded ^13^C is obtained from fitting. (c) Measured and best‐fit simulation of the REDOR dephasing of A22. The measured *S*/*S*_0_ value decays below ~0.9, indicating that only one of the five ETM monomers is in close contact with the drug. Thus, simulated REDOR intensities are scaled by 20%. (d) Measured REDOR dephasing of the F20/F23 peak along with best‐fit REDOR simulations.

To complement these ^13^C–^19^F and ^13^C–^15^N distance restraints, we further measured ^1^H–^19^F distances between protein H^N^ and the hexamethylene fluorines using a 2D hNH‐resolved ^1^H–^19^F REDOR experiment (Figure 7). The ^15^N‐labeled and perdeuterated ETM gives rise to resolved 2D ^1^H–^15^N correlation spectra (Figure 7a). Application of the ^19^F pulses yielded REDOR difference signals for H^N^ sites that are close to the hexamethylene fluorines. At a mixing time of 3.37 ms, we observed the largest difference intensities for A22 and F20/F23, indicating that the hexamethylene ring lies in the middle of the TM peptide. Additional weaker intensities are observed for V24, F26, T30, and A32. We simulated the measured ^1^H–^19^F REDOR dephasing (*S*/*S*_0_) using a three‐spin system that includes a protein H^N^ and the two fluorines (Figure 7b, Figure S3d), and parameterized the two H–F distances by the distance *d*_CH_ between the protein H^N^ and the fluorine‐bonded carbon. Using a 20% intensity scaling to account for the stoichiometry of one HMA per pentamer, we obtained distances of 8.6–8.9 Å from the HMA fluorines to the amide protons of A22 and F20/F23.

### 2.3 Distance‐constrained docking of HMA to ETM pentamers

On the basis of these distance restraints for the protein–drug complex, we docked HMA to the structure of the open ETM (Medeiros‐Silva et al., 2023). Since the protein‐detected REDOR data indicates that the drug is not equidistant to all five helices, we evaluated two scenarios of the drug location relative to the pentamer. In one scenario, two neighboring helices provide the atomic contacts, while in the second scenario, all close contacts originate from a single helix. The second scenario is less likely, as it implies that all five helices bind HMA, which would result in a binding stoichiometry of 5:5, which is inconsistent with the data. For each scenario, we implemented two docking procedures: unambiguously restrained docking requires all distance restraints to be simultaneously satisfied by each drug whereas ambiguously restrained docking allows the drug to satisfy only a subset of the distance restraints. During this ambiguous docking, each restraint is imposed 50% of the time.

**FIGURE 8:**
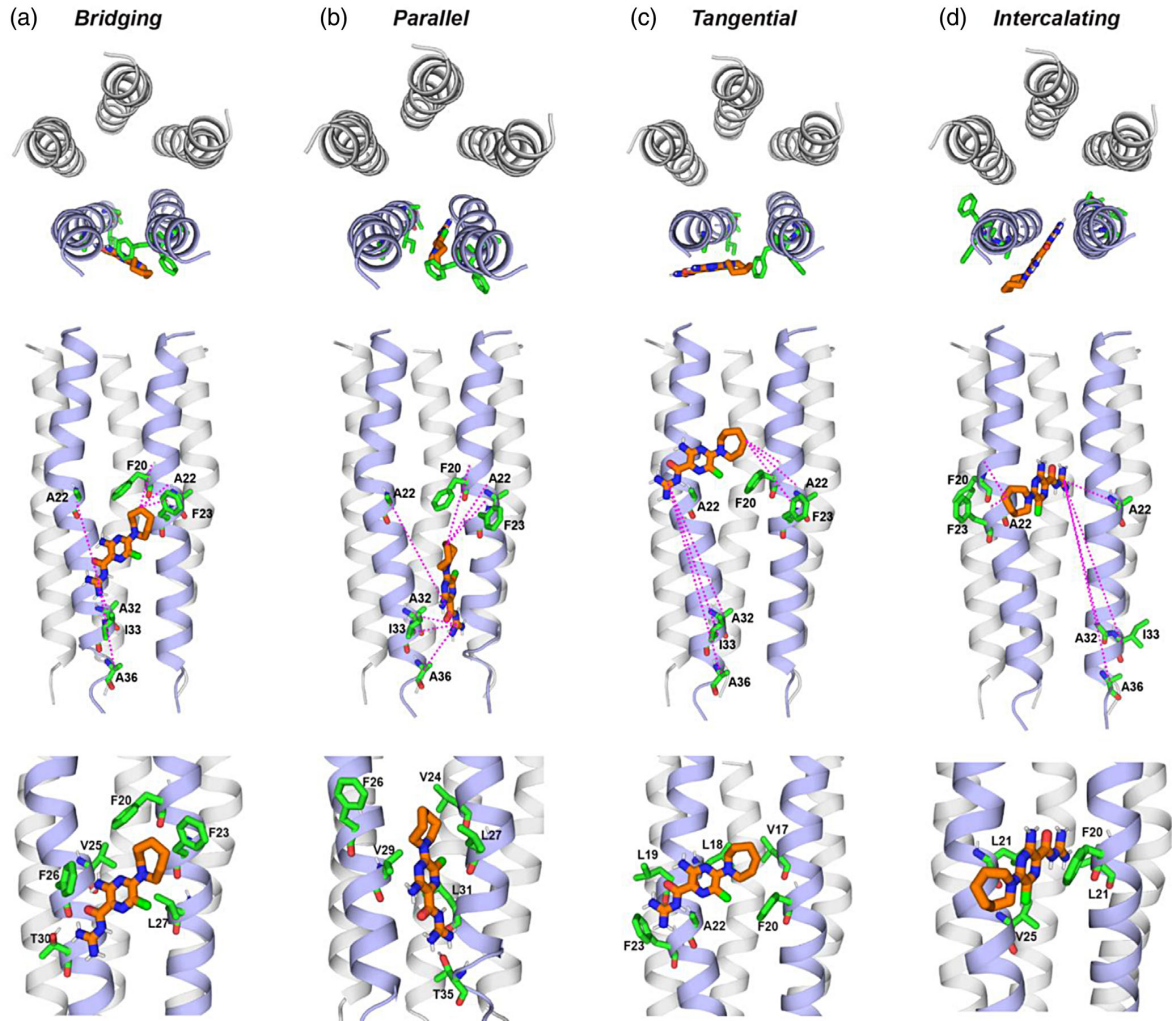
Distance‐restrained docking of HMA (orange) to the ETM pentamer at acidic pH. The protein structure is that of the low‐pH open state structure (Medeiros‐Silva et al., 2023). Four lowest‐energy poses are shown in N‐terminal view (top row), a side view with key measured distance restraints (middle row), and another side view showing HMA‐interacting sidechains (bottom row). For clarity, the two helices in the front are colored in purple while the three helices in the back are shown in gray. (a) Lowest‐energy pose from unambiguously restrained docking. HMA bridges two helices on the lipid‐facing surface of the pentamer. The guanidinium group points to T30 of one helix whereas the hexamethylene ring occupies an aromatic pocket formed by F20 and F23. (b) The second lowest‐energy pose from unambiguously restrained docking. HMA is parallel to two neighboring helices. The guanidinium points to the C‐terminus whereas the hexamethylene ring points to the N‐terminus. (c) Lowest‐energy pose from ambiguously restrained docking. HMA adopts a tangential orientation; the guanidinium interacts with the peptide backbone while the hexamethylene ring contacts hydrophobic residues such as V17, L18, and F20. (d) The second lowest‐energy pose from ambiguously restrained docking. HMA intercalates between two neighboring helices; the guanidinium interacts with F20 while the hexamethylene ring is exposed to lipids. Ambiguous restrained docking results in poses in which the HMA long axis is perpendicular to the TM helix.

We first assigned the drug–protein distances to two neighboring helices in the pentamer. The ^13^C–^15^N and ^13^C–^19^F distance constraints to the guanidinium group are assigned to one helix, whereas the ^1^H–^19^F distance constraints involving the hexamethylene ring are assigned to the neighboring helix. The unambiguously restrained docking resulted in a lowest‐energy “bridging” pose that straddles two neighboring helices in the C‐terminal half of the TM domain (Figure 8a). The long axis of the drug is tilted with respect to the bilayer normal whereas the plane of the drug is approximately tangential to the periphery of the pentamer. The polar guanidinium points to the C‐terminus of the protein, interacting with T30 of chain A (T30_A_). The pyrazine ring interacts with F26 of the same chain, whereas the nonpolar hexamethylene occupies the hydrophobic pocket formed by V25_A_, F20_B_, and F23_B_. In this bridging pose, all distance restraints are satisfied except for the ^13^Cα–^15^N distances to A22_A_ and A36_A_.

The second lowest‐energy pose from the unambiguously restrained neighboring‐helix docking shows a parallel orientation of the long axis of the drug with respect to the bilayer normal (Figure 8b). HMA is positioned between two helices, with the polar guanidinium pointing to the C‐terminus, interacting with T35 of chain B, whereas the pyrazine and hexamethylene moieties interact with residues V29_A_, V24_B_, L27_B_, and L31_B_. This parallel pose satisfies most of the measured restraints, except for the Cα‐N distances to A22_A_ and A36_A_, which are too far from the guanidinium compared to the measured ^13^C–^15^N distances.

Because the lowest‐energy poses from the unambiguous restrained docking did not satisfy all measured restraints, we tested ambiguous docking to assess if alternative binding modes exist. Interestingly, the lowest energy pose (Figure 8c) shows the drug to shift up toward the N‐terminus, with the guanidinium group interacting with the A22_A_ and F23_A_ backbone and the F23_A_ sidechain. The pyrazine and hexamethylene groups interact with residues L19_A_, L18_A_, V17_B_, and F20_B_. The plane of the drug is tangential to the pentamer surface whereas the long axis of the molecule is almost perpendicular to the TM helices. When the distance restraints for the guanidinium and hexamethylene are assigned to neighboring helices in the opposite direction, the lowest‐energy pose intercalates the drug into the helix interface (Figure 8d). The pyrazine ring participates in *π*–*π* stacking with the F20 ring from one helix whereas the hexamethylene group is exposed to lipids.

## 3 DISCUSSION

### 3.1 Consistency of the distance‐restrained HMA binding site with the chemical shift data

The solid‐state NMR data shown here represent the first direct distance‐based investigation of the HMA binding site in the TM domain of the SARS‐CoV‐2 E protein in lipid bilayers. Four protein–drug contact experiments were conducted, measuring ^19^F–^19^F, ^19^F–^13^C, ^13^C–^15^N, and H^N^–^19^F proximities and distances. Except for the ^19^F–^19^F spin diffusion experiment, the other three measurements use the REDOR technique, which gives both quantitative distances and the binding stoichiometry of the drug to the protein. These data consistently show that HMA binds to residues in the middle of the TM domain on the lipid‐facing surface. This result is unexpected, because previous CSP data showed the largest changes for N‐ and C‐terminal residues (Li et al., 2014; Mandala et al., 2020; Park et al., 2021; Pervushin et al., 2009). However, CSPs measured on one of the samples used here for distance experiments qualitatively reproduced the previous trend (Figure 4), indicating that HMA contact with the central TM residues does not contradict the large CSPs for the terminal residues. This implies that HMA binding to the middle of the TM domain allosterically induces conformational changes to the N‐ and C‐termini. In addition to the terminal residues, the central TM residues show larger CSPs than the surrounding residues, which had not been observed before. Since all distance measurements here are conducted at P:D molar ratios of 5:5 or 5:2 (Table S1) whereas all previous CSP measurements were conducted under larger excess of drug (P:D = 5:50), low drug concentrations are required to reveal the substantial CSPs for the middle of the TM domain.

At the P:D ratios of 5:2 and 5:5, the drug‐detected (Figure 5) and protein‐detected (Figures 6 and 7) REDOR data indicate that one HMA binds every five ETM helices, and binding is asymmetric, with one of the five helices lying the closest to a functional group of the drug. The drug‐detected ^13^C–^19^F REDOR data (Figure 5d) show that the REDOR dephasing plateaued to ~50% when the sample contains two equivalents of HMA for five helices (P:D = 5:2), while the dephasing plateaued to ~80% when the sample contains five equivalents of HMA for five helices (P:D = 5:5). These results indicate that the binding stoichiometry is one HMA for five helices. At the same time, protein‐detected ^1^H–^19^F and ^13^C–^15^N REDOR data (Figures 6f and 7c) show that only 20% of the protein is dephased by the drug. Thus, the one equivalent of bound drug is not equidistant to all five helices.

In principle, two models can explain these data. In the first model, 20% of the helical bundles have five bound drugs on the lipid‐facing surface, whereas the remaining 80% of the helical bundles do not bind any HMA. This model can be ruled out for two reasons. First, the coexistence of two dramatically different types of pentamers should give rise to two sets of protein chemical shifts, which are not observed. Second, biochemical studies indicate an IC_50_ of ~10 μM for HMA, and the active protein species is believed to be a pentamer. Therefore, under the ~100 mM concentration of the protein in the solid‐state NMR samples, when HMA is at 2‐ or 5‐fold excess relative to the pentamer (P:D = 5:2 or 5:5), all physiologically relevant sites should be saturated.

In the second model, one HMA binds each ETM pentamer, and the bound HMA is not centrally located in the channel pore but instead lies on the lipid‐facing surface of the protein, closest to one of the helices. This model is consistent with all the measured data. Since the one bound HMA can interact with two neighboring helices, this interaction, when sufficiently perturbative, might prevent two more helices from interacting with another HMA. We used low drug concentrations relative to the protein in this study in order to observe the highest affinity binding mode. It is possible that at higher HMA concentrations, additional binding sites may be occupied. The low‐pH and high‐pH ETM samples show consistent REDOR results, indicating that the binding mode is relatively insensitive to pH. Thus, pore diameter differences between the closed and open states (Medeiros‐Silva et al., 2022; Medeiros‐Silva et al., 2023) do not cause pronounced changes to the mode of interaction of HMA with the protein.

We detected only chemical shift perturbation and not peak doubling, despite the fact that the one equivalent of drug binds asymmetrically to each pentamer. This can be explained by the five helices exchanging between apo and HMA‐bound conformations at a faster rate than the ^1^H chemical shift difference of ~0.1 ppm, which corresponds to 60 Hz or ~380 s^−1^. This limiting exchange rate is modest, and may be achieved by HMA diffusing among the five helices of each pentamer from the membrane side.

HMA‐induced CSPs had been reported for a number of E constructs before, including E(8–38) (Mandala et al., 2020; Pervushin et al., 2009), E(1–38) (Park et al., 2021), E(8–65) (Li et al., 2014), and E(1–75) (Toft‐Bertelsen et al., 2021). These constructs were reconstituted in a variety of detergent micelles (Li et al., 2014; Park et al., 2021; Pervushin et al., 2009; Toft‐Bertelsen et al., 2021) as well as in lipid bilayers (Mandala et al., 2020). None of these sample differences changed the qualitative trend of the CSPs. The only common feature in all these CSP measurements was that most samples contained a larger excess of drug, with a P:D of 5:50. In a recent ssNMR study of HMA‐bound ETM (Mandala et al., 2020), the CSPs were found to be small at a P:D of 5:5 and became substantial after the drug concentration increased to reach a P:D of 5:50. Therefore, the large CSPs at the N‐ and C‐termini occur at high HMA concentrations, whereas the surface‐binding sites are occupied at low HMA concentrations. Together, these data indicate that HMA has higher affinity for the lipid‐facing residues in the middle of the TM domain than for the pore‐facing polar residues at the two termini of the TM channel.

### 3.2 Comparison of HMA binding to E with other small‐molecule binding to viroporins

The high affinity of HMA for the lipid‐facing residues of ETM qualitatively differs from the amantadine binding mode to the influenza A M2 (AM2) protein (Hong & DeGrado, 2012). AM2 also contains two binding sites for amantadine: a site inside the N‐terminal channel pore near a crucial S31 (Stouffer et al., 2008), and a site on the lipid‐facing surface of the protein at D44 and R45 (Schnell & Chou, 2008). M2‐amantadine ^13^C–^2^H REDOR distance measurements as a function of P:D ratios showed that the first equivalent of amantadine binds inside the N‐terminal pore and occludes the channel (Andreas et al., 2013; Cady et al., 2010; Pielak et al., 2011), whereas excess drugs bind to the protein–lipid interface (Cady, Wang, & Hong, 2011; Cady, Wang, Wu, et al., 2011). Therefore, this canonical viroporin is an example where the water‐filled channel pore provides the high‐affinity binding site for the drug whereas the lipid‐facing surface forms a non‐specific binding site.

Despite this precedent, the chemistry and structures of amantadine and HMA differ, cautioning against a direct translation of the M2‐amantadine binding mode to E‐HMA binding. HMA has an extended polar acyl guanidinium and pyrazine ring whereas amantadine has no appreciable polar functionality except for the NH_2_ group. SARS‐CoV‐2 ETM has an uninterrupted hydrophobic segment from residue V17 to V29, whereas the TM domain of AM2 is punctuated by multiple polar residues at S23, S31, H37, D44, and R45. Because the hydrophobic environment of ETM inside and outside the pore is not substantially different, small‐molecule binding may be dictated by chemical and conformational features other than hydrophobicity. We hypothesize that an important factor for HMA binding may be the aromatic belt in the middle of the ETM helical bundle. This aromatic belt consists of three regularly spaced Phe residues, F20, F23, and F26. Recent measurements of the water and lipid contact of these Phe residues showed that these Phe sidechains adopt two rotameric conformations, whose populations change between the closed and open states (Medeiros‐Silva et al., 2022). In the neutral‐pH closed state, the Phe conformational equilibrium shifts towards pore‐facing, while in the acidic‐pH open state, the Phe sidechain conformations shift towards lipid‐facing. The distance‐constrained docking shows that the pyrazine and azepane moieties of HMA interact with these Phe residues (Figure 8). Among the lowest‐energy docked poses, the interhelical bridging pose places the hexamethylene ring against both F20 and F23 of the same helix (Figure 8a). Similarly, the tangential and intercalating docking poses stack the hexamethylene ring against these Phe residues (Figure 8c,d). These structural features suggest an inhibition mechanism in which HMA interacts with the lipid‐facing Phe sidechains to either disrupt the functional *π*–*π* interactions or prevent the conformational motion that is required to activate the channel. In other words, HMA may inhibit ETM by preventing the aromatic belt from loosening the helical bundle using suitable conformational changes.

Additional evidence that HMA may target the lipid–protein interface rather than the N‐terminal pore for inhibition is suggested by the amino acid sequences of the envelope proteins of related viruses (Figure 1d). HMA has been shown to inhibit the ion current of the E protein of not only SARS‐CoV‐2 but also human coronavirus 229E (hCoV‐229E), mouse hepatitis virus (MHV) (Wilson et al., 2006), as well as the channel activity of HIV‐1 Vpu (Ewart et al., 2002; Ewart et al., 2004; Zumla et al., 2016). In contrast, HMA does not inhibit the E protein of avian infectious bronchitis virus (IBV) (Wilson et al., 2006). The SARS‐CoV‐2, hCoV‐229E, and MHV E proteins all have a polar Asn or Gln residue at position 15, but the HMA‐inhibited HIV‐1 Vpu lacks a polar residue at this position and instead has a Val. The IBV E protein lacks an Asn or Gln at this position and instead replaces it with a Thr. Like SARS‐CoV‐2, hCoV‐229E, MHV, and IBV envelope proteins all possess negatively charged residues at the N‐terminus, whereas HIV‐1 Vpu does not. This comparison indicates that the polar and charged residues in the N‐terminal region of these proteins do not correlate with their inhibition by HMA. Instead, the TM region of all HMA‐inhibited proteins is hydrophobic, while the TM region of the noninhibited IBV viroporin is less hydrophobic, containing several Tyr and Gly residues. This sequence difference supports the hypothesis that the hydrophobic lipid‐facing surface of these viroporins may be the primary site of inhibition by HMA.

### 3.3 Comparison with other membrane proteins that bind small molecules from the lipid side

Increasing structural information has recently shown that many small‐molecule drugs associate with the lipid‐facing surface of G protein‐coupled receptors, ion channels, transporters, and membrane‐bound enzymes (Payandeh & Volgraf, 2021). One example is the P2Y_1_ receptor antagonist BPTU, which is bound to a membrane‐exposed extrahelical site of the receptor (Zhang et al., 2015). The urea group of BPTU coordinates to the protein while the hydrophobic portion of the drug is exposed to the lipids. Importantly, the lipophilic substituents of these compounds are critical for potency. Similar results have been obtained for ion‐channel targeting drugs, such as the transient receptor potential ankyrin 1 (TRPA1) antagonist GDC‐0334 (Balestrini et al., 2021; Chen et al., 2018). This molecule was shown by cryo‐EM to be bound in a shallow intrahelical pocket, with the polar proline sulfonamide group buried in the pocket and the rest of the drug exposed to the membrane. Attempts to reduce the lipophilicity of this antagonist while retaining activity were unsuccessful, indicating that the lipophilicity is necessary for function. HMA has many similarities to these compounds. It is amphipathic, with the hydrophobic hexamethylene ring being crucial for efficacy, as amiloride alone does not inhibit the channel activity (Park et al., 2021; Pervushin et al., 2009). This suggests that the hexamethylene moiety, by increasing the lipophilicity, may facilitate the approach of the drug to the protein from the membrane side. Once in contact with the aromatic belt, HMA may prevent the necessary conformational rearrangement of the Phe residues to open the channel. At the same time, the guanidinium moiety might interact with the lipid phosphate headgroups through salt bridge interactions and hydrogen bonding (Mani et al., 2006; Su et al., 2009; Tang et al., 2007) to stabilize HMA in the membrane. In the two unambiguously restrained docking poses, HMA aligns approximately vertically, pointing the guanidinium group to the membrane‐water interface, consistent with this model.

The current results do not exclude a potential second binding mode of HMA inside the pore: at higher drug concentrations, the bulky hexamethylene group might facilitate physical occlusion of the channel pore. The current study focuses on the TM portion of the E protein. Addition of the first seven residues of the protein outside the membrane could affect the drug binding equilibria and might promote N‐terminal pore binding. Future studies should investigate the inhibitory effects of HMA at different concentrations and on difference E constructs, to elucidate which binding mode is chiefly responsible for channel inhibition. Finally, because of the chemical versatility of the amiloride moiety for substitution by a large number of functional groups, amiloride analogs have recently been shown to bind conserved stem loops in the untranslated 5″ end of viral RNA to reduce virus replication (Zafferani et al., 2021). Elucidating the structures of amiloride‐protein and amiloride‐RNA complexes in SARS‐CoV‐2 is thus important for gaining insights into the general chemical and structural principles of amilorides as antiviral drugs.

## 4 MATERIALS AND METHODS

### 4.1 Expression and purification of ^13^C, ^15^N labeled ETM

We expressed and purified ^13^C, ^15^N‐labeled ETM, ^13^C‐only labeled ETM, and ^13^C, ^15^N, ^2^H‐labeled ETM (residues 8‐38) (Figure 1a) using previously described protocols (Mandala et al., 2020; Medeiros‐Silva et al., 2022). Briefly, *E. coli* BL21 (DE3) cells were transformed with a His_6_‐SUMO‐ETM fusion protein gene. Cells were grown at 37°C in M9 media containing ^13^C‐labeled D‐glucose and suitably labeled NH_4_Cl. Protein expression was induced with IPTG. Cells were harvested, suspended in lysis buffer and treated with lysozyme, Triton‐X, and benzonase nuclease. Cells were lysed by sonication on ice, then the cell debris was removed, and the supernatant was purified using a Ni^2+^ affinity column. Eluted His_6_‐SUMO‐ETM was cleaved with SUMO protease and tris(2‐carboxyethyl)phosphine, and the resulting mixture was purified by reverse‐phase HPLC to obtain ETM. The yield of ETM was about 14 mg per liter of M9 culture.

### 4.2 Synthesis of V14F‐CF_3_ ‐labeled ETM

V14F‐CF_3_ labeled ETM (residues 8–38) was synthesized using Fmoc solid phase chemistry. The peptide replaces the V14 residue with 4‐CF_3_‐labeled Phe (ChemImpex). ^13^C, ^15^N‐labeled G10, I13, and S16 were also incorporated into the peptide (Figure 1a). The peptide was synthesized on the 0.15 mmol scale using a custom‐built rapid‐flow peptide synthesizer (Simon et al., 2014). H‐Rink amide ChemMatrix resin (0.075 mmol, 0.15 g at 0.5 mmol/g loading size) was loaded into the reactor, which was kept in a 70°C water bath during synthesis. Amino acids were dissolved in hexafluorophosphate azabenzotriazole tetramethyl uranium (HATU) solution in 0.57 M dimethylformamide (DMF) (2.5 mL per residue, 9.5 equiv). Immediately before each coupling, N,N‐diisopropylethylamine (261 μL, 1.5 mmol, 20 equiv) was added to each amino acid. Unlabeled or ^19^F‐labeled amino acids were coupled with 10‐fold excess for 40 s, while ^13^C, ^15^N‐labeled amino acids were coupled with 4‐fold excess. To reduce single‐residue deletion impurities, double couplings were performed at G10, I13, V14F, ^18^LLFL^21^, ^23^FVVFLL^28^, and R38. Each coupling was followed by a 65 s wash step (DMF, 20 mL/min). After the first wash, Fmoc deprotection was performed with a 20% piperidine solution flowing at 20 mL/min for 25 s. The reactor was washed again with DMF for 65 s at 20 mL/min. After the final coupling, the resin was washed with DMF for 5 min at 20 mL/min. The resin was then washed 3 times with dichloromethane and dried under house vacuum overnight. The peptide was deprotected and cleaved from the resin using 7.5 mL trifluoroacetic acid/phenol/water/triisopropylsilane solution (88:5:5:2 by volume) for 2 h. The resin was filtered off, and the crude peptide was precipitated from the cleavage solution with cold diethyl ether and then washed twice with cold diethyl ether before being dried under vacuum overnight at room temperature. The resulting crude peptide was dissolved in trifluoroethanol (TFE) and purified by preparative reverse‐phase HPLC using a Vydac C4 column (22 mm × 250 mm, 10 μm particle size) and a linear gradient of 80%–100% methanol (channel B) over 25 min at a flow rate of 10 mL/min (channel A is water). The peptide was eluted at ∼99% methanol. Fractions containing the peptide were assessed for relative purity by MALDI‐MS (theoretical MW: 3490.5 Da; experimental: 3486.4 Da). Fractions assessed to contain pure peptide were pooled and lyophilized. About 32 mg of pure peptide was obtained, corresponding to an overall yield of 12%.

### 4.3 Synthesis of fluorinated HMA F_2_‐HMA

F_2_‐HMA was synthesized according to the literature (Buckley et al., 2018; Murai et al., 2015). Briefly, to a suspension of methyl 3‐amino‐5,6‐dichloro‐2‐pyrazinecarboxylate (444 mg, 2.0 mmol) in 2‐propanol (4 mL) were added 4,4‐difluoroazepane hydrochloride (1 g, 5.8 mmol) and diisopropylethylamine (2.01 mL, 11.6 mmol). The reaction mixture was heated at reflux for 2 h and stirred overnight at room temperature. The volatiles were removed by rotary evaporation and the residue was purified by silica gel column chromatography (hexanes/ethyl acetate = 0%–100%) to give a yellow solid (314 mg, 49%, m/z = 321.4). Next, a methanolic solution of guanidine was prepared by addition of NaOMe (25% solution in methanol, 18.7 mmol) to guanidine HCl (1.64 g, 17.2 mmol) in anhydrous methanol (6 mL) at room temperature. After stirring for 20 min, the solid was filtered through a fritted syringe and the clear filtrate was added to a solution of methyl ester (1.1 g, 3.4 mmol) in anhydrous MeOH (6 mL). The reaction mixture was heated at 85°C for 2 h and cooled to room temperature. The reaction mixture was neutralized by addition of 1 M HCl solution. After lyophilization, the crude product was purified by RP‐HPLC to give a yellow solid (844 mg, 54%, m/z = 348.3).

### 4.4 Synthesis of guanidinium ^13^C, ^15^N‐labeled HMA

**SCHEME 1:**
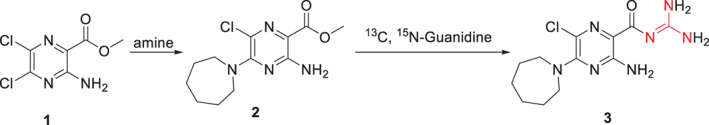
Compound **2** was synthesized following the reported procedures (Cragoe et al., 1967).

Chemicals were ordered from commercial sources and used without further purification. Synthesis procedures for reactions are shown in Scheme 1. ^1^H and ^13^C NMR spectra were recorded on a Bruker 400 MHz spectrometer. Chemical shifts are reported in parts per million (ppm) referenced to the residual solvent CDCl_3_ peak at 7.26 ppm on the internal standard tetramethylsilane (TMS) at 0.00 ppm. The following abbreviations were used in reporting spectra: s, singlet; d, doublet; t, triplet; q, quartet; m, multiplet; dd, doublet of doublets. All reactions were carried out under N_2_ atmosphere unless otherwise stated. HPLC‐grade solvents were used for all reactions. Low‐resolution mass spectra were obtained using an ESI technique on a 3200 Q Trap LC/MS/MS system (Applied Biosystems). Compound purity was assessed using Shimadzu LC‐MS with Waters XTerra MS C‐18 column (part #186000538), 50 × 2.1 mm, at a flow rate of 0.3 mL/min; *λ* = 250 and 220 nm; mobile phase A, 0.1% formic acid in H_2_O, and mobile phase B′, 0.1% formic in 60% isopropanol, 30% CH_3_CN and 9.9% H_2_O.

A solution of ^13^C, ^15^N‐guanidine.HCl (0.5 mmol) in DMF (5 mL) was cooled with ice batch and t‐BuOK (1.1 mL, 1 N in THF) was added. After 5 min, compound **2** (0.6 mmol) was added in one portion. The reaction was heated to 60°C and stirred overnight. After removing solvent, the residual was purified with reverse‐phase HPLC to give the target product **3** as a white solid. Yield: 38%. ^1^H NMR (500 MHz, CD_3_OD) *δ* 3.96–3.90 (m, 4H), 1.89–1.83 (m, 4H), 1.64–1.57 (m, 4H). ^13^C NMR (125 MHz, CD_3_OD) *δ* 165.4, 165.3, 154.3, 153.5, 119.1, 51.0, 28.0, 26.2. C_11_^13^CH_19_ClN_4_^15^N_3_O ESI‐MS: m/z (M+H^+^): 316.1 (calculated), 316.1 (found) (Figure S1).

### 4.5 Preparation of proteoliposomes

We prepared a total of 13 membrane samples containing both the protein and the drug, and additional control samples containing the protein alone or drug alone. For the protein/drug mixtures, we co‐solubilized ETM, HMA and lipids in organic solvents. All samples used a model membrane containing 1,2‐dimyristoyl‐sn‐glycero‐3‐phosphocholine (DMPC) and 1,2‐dimyristoyl‐sn‐glycero‐3‐phospho‐(1′‐rac‐glycerol) (DMPG) at a molar ratio of 7:3 or 8:2. For all intermolecular contact measurements, the P:D molar ratio ranged from 5:5 to 5:2 (Table S1). We express these P:D molar ratios in units of 5 protein monomers because E forms pentamers in lipid bilayers (Somberg et al., 2022), so that the P:D ratios can be readily converted to the molar ratio of channels to drug. At a P:D of 5:2, the sample contains two equivalents of HMA to each pentamer. This drug amount is much lower than that in most literature studies of HMA induced chemical shift perturbation to E, where the P:D was 5:50. At the low drug concentrations used in the current studies, nonspecific binding effects are expected to be minimal. The protein monomer/lipid molar ratios range from 5:100 to 5:50.

We dissolved ETM in either TFE or methanol at ~1 mg/mL, HMA in TFE or DMSO at ~30 mg/mL, and lipids in chloroform at ~5 mg/mL. We chose methanol instead of TFE to dissolve fluorinated protein samples (V14F‐CF_3_‐ETM) in order to avoid a large ^19^F peak from residual TFE solvent in the spectrum. Similarly, we chose DMSO to dissolve F_2_‐HMA in order to avoid the solvent ^19^F signal. The peptide, lipid, and drug solutions were combined to give a homogeneous and transparent solution. The final concentrations were about 0.3 mg/mL for the peptide, 2 mg/mL for the lipid, and 0.1 mg/mL for the drug. The bulk organic solvent was removed with a stream of nitrogen gas (RT, 1 h). Residual organic solvent was further removed under house vacuum (50 mBar, RT, 4 h), followed by lyophilization overnight. The dry proteoliposome film was resuspended in 3 mL of pH 7.5 Tris buffer (25 mM Tris, 25 mM NaCl, 1 mM EDTA, 0.07 mM NaN_3_) or in 3 mL of pH 4.5 acetate buffer (25 mM acetate, 25 mM NaCl, 0.07 mM NaN_3_). We have shown recently that the pH 4.5 environment increased the water accessibility of the protein and the conformational disorder of the termini compared to the pH 7.5 sample (Mandala et al., 2020; Medeiros‐Silva et al., 2022). Ca^2+^ produced the same effect as acidic pH, thus it is omitted in this study to simplify the membrane sample preparation. We denote the pH 4.5 sample as the open state and the pH 7.5 sample as the closed state of ETM in this study.

The proteoliposome suspension was vortexed and sonicated five times (2 s each), then incubated for 1 h at room temperature with gentle agitation every 10 min. The homogeneous solution was frozen in liquid nitrogen until solid (90 s) and thawed in a 42°C water bath until warmed (4 min). This freeze–thaw cycle was repeated 8 times to produce multilamellar vesicles, then the vesicle solution was ultracentrifuged at 311,000 × *g* at 10°C for 4 h to obtain a membrane pellet. Most pellets are opaque off‐white, except the F_2_‐HMA containing samples, which are bright yellow. The wet pellet was dried in a desiccator at RT until the sample reached a hydration level of ∼40% (w/w) water with respect to the total mass of the pellet. The pellet was packed into the appropriate MAS rotor by spinning at 5000 × *g* using a benchtop Beckman Coulter swinging‐bucket rotor.

### 4.6 Solid‐state NMR spectroscopy

All magic‐angle‐spinning (MAS) solid‐state NMR experiments were carried out on Bruker AVANCE spectrometers ranging from 900 MHz (21.1 T) to 400 MHz (9.4 T) ^1^H Larmor frequencies. ^13^C and ^15^N spectra were measured on an 800 MHz (18.8 T) spectrometer using a Bruker 3.2 mm HCN probe or a BlackFox 3.2 mm HCN probe. Additional ^13^C and ^15^N spectra were measured on a 900 MHz (21.1 T) spectrometer using a 3.2 mm Efree HCN probe. ^19^F experiments were conducted on a 600 MHz (14.1 T) spectrometer using a 1.9 mm HFX probe and a 400 MHz (9.4 T) spectrometer using a 4 mm HFX probe.

^13^C chemical shifts were referenced externally to the adamantane CH_2_ signal at 38.48 ppm on the TMS scale. ^15^N chemical shifts were referenced externally to ^15^N‐acetylvaline at 122.0 ppm on the liquid ammonia scale. ^19^F chemical shifts were referenced externally to the 5‐^19^F‐trpytophan signal at −122.1 ppm on the CF_3_Cl scale. ^1^H chemical shifts were referenced internally to the DMPC Hγ peak at 3.264 ppm on the TMS scale. Unless otherwise specified, all indicated temperatures refer to the sample temperatures, which were estimated from the bulk water ^1^H chemical shift using the equation *T* (K) = 96.9 × (7.83 ppm − *δ*_H2O_) (Böckmann et al., 2009). Typical radiofrequency (rf) field strengths were 50–90 kHz for ^1^H, 50–60 kHz for ^13^C, 30–40 kHz for ^15^N and 50–80 kHz for ^19^F.

2D ^13^C–^13^C correlation spectra were measured with 23 ms combined R2_*n*_^ν^‐driven (CORD) mixing (Hou et al., 2013) under 10.5 kHz MAS at a sample temperature of 274 K on the 800 MHz spectrometer. 1D NHHC spectrum (Lange et al., 2002) was measured under 11.8 kHz MAS at 277 K on the 900 MHz NMR. The cross polarization (CP) contact times were 1 ms for ^1^H–^15^N, 1 ms for ^15^N–^1^H, and 800 μs for ^1^H–^13^C CP. NHHC spectra were measured using ^1^H mixing times of 0.5 ms and 1 ms and were coadded. ^13^Cα‐^15^N frequency‐selective REDOR experiments (Figure S2a) (Gullion & Schaefer, 1989; Jaroniec et al., 2001) were conducted on the 800 MHz spectrometer under 10.5 kHz MAS at 274 K. ^13^Cα signals were selected using a ^13^C Gaussian 180° pulse length of 380.95 μs, which corresponds to 4 rotor periods, centered at 58.0 ppm. Two ^13^C spectra were measured for each REDOR mixing time, a control spectrum (*S*_0_) without ^15^N pulses and a dephased spectrum (*S*) with two ^15^N 180° pulses per rotor period. The ^15^N rf field strength for the REDOR *S* experiment was 35.7 kHz. The ^13^C–^15^N REDOR mixing times were 12 ms (126 rotor periods) and 20 ms (210 rotor periods). ^1^H two‐pulse phase‐modulation (TPPM) (Bennett et al., 1995) decoupling at 83.3 kHz was applied during the REDOR period.

All ^19^F NMR experiments were conducted on the 600 MHz spectrometer using a 1.9 mm HFX probe. The 2D hNH‐resolved ^1^H–^19^F REDOR experiments (Shcherbakov et al., 2019) were conducted under 38 kHz MAS at 265 K (Figure S2b). ^15^N WALTZ‐16 decoupling (Shaka et al., 1983) was applied during the REDOR period, and the MISSISSIPPI sequence (Zhou & Rienstra, 2008) at a ^1^H rf field strength of 15 kHz was used to suppress the water ^1^H signal. ^13^C–^19^F REDOR experiments (Figure S2c) of ^13^C‐labeled HMA and CF_3_‐V14F‐labeled ETM were conducted under 10.5 kHz MAS at 260 K. A ^1^H TPPM decoupling field of 83.3 kHz was applied during the REDOR mixing period. This ^13^C–^19^F REDOR experiment was broadband, since HMA is singly labeled with ^13^C, thus obviating the need for ^13^C–^13^C J decoupling. 2D ^19^F–^19^F correlation spectra were measured under 25 kHz MAS using 300 ms CORD spin diffusion. More detailed experimental parameters are given in Table S2.

^1^H‐detected 2D hNH and 3D hCANH correlation spectra were measured on the 600 MHz NMR using a 1.3 mm HCN probe. Residue‐specific CSPs were calculated from the measured ^1^H, ^13^C, and ^15^N chemical shifts (*δ*) according to (Williamson, 2013):

$$∆\delta =\sqrt{{\left(\delta_{H}^{\mathrm{HMA}}-\delta_{H}^{\mathrm{apo}}\right)}^{2}+0.30\;{\left(\delta_{C}^{\mathrm{HMA}}-\delta_{C}^{\mathrm{apo}}\right)}^{2}+0.14{\left(\delta_{N}^{\mathrm{HMA}}-\delta_{N}^{\mathrm{apo}}\right)}^{2}}$$

### 4.7 NMR spectral analysis

All NMR spectra were processed in the TopSpin 3.6 software. One‐dimensional (1D) spectra were typically processed using Gaussian apodization with LB = −30 Hz and GB = 0.03, while 2D spectra were typically processed using QSINE apodization with SSB = 3.

REDOR dephasing values (*S*/*S*_0_) were extracted from peak heights. For ^13^C–^15^N and ^13^C–^19^F REDOR data, error bars (*σ*) were estimated from the signal‐to‐noise ratio (SNR) of the *S*_0_ spectrum according to $\sigma =\frac{S}{S_{0}}{\left[\mathrm{SN}R^{-2}+{\left(\mathrm{SNR}\times S/S_{0}\right)}^{-2}\right]}^{1/2}$. For the low‐pH hNH‐resolved ^1^H–^19^F REDOR data, *S*_0_ and *S* experiments were conducted in 3‐h blocks. This block‐averaging minimizes fluctuations in the ^1^H–^15^N CP efficiency, rf power levels, and solvent suppression. For these experiments, presented error bars are the standard deviation *σ* of the measured *S*/*S*_0_ values of the experimental replicates. Best‐fit simulations were obtained from the minimum $\chi_{\nu }^{2}=\frac{1}{\nu }\sum {\left(\exp-\mathrm{sim}\right)}^{2}/\sigma^{2}$, where *exp* and *sim* are the experimental and simulated *S*/*S*_0_ values, respectively, and *ν* is the number of degrees of the freedom in the fit. In this case, *ν* equals the number of data points minus one. The distance value giving the minimum $\chi_{\nu }^{2}$ for a given stoichiometry is taken as the best‐fit distance, while the distance range corresponds to distances for which $\chi_{\nu }^{2}<\chi_{\nu ,\min}^{2}+1$.

### 4.8 REDOR simulations

All REDOR simulations were conducted using the SIMPSON software (Bak et al., 2000) hosted on NMRbox (Maciejewski et al., 2017). Simulated curves were fit to experimental data in MATLAB R2021b. ^13^C–^19^F REDOR simulations between the HMA guanidinium ^13^C and protein CF_3_ were conducted using a two‐spin model (Figure S3a) in which the three fluorines of CF_3_ were modeled as a pseudo‐fluorine atom with 1.73 times the gyromagnetic ratio of ^19^F (Elkins et al., 2017). Literature ^19^F CSA tensor values for CF_3_ are used (Roos et al., 2018). To simulate the ^13^C–^15^N_3_ REDOR curves between protein ^13^Cα and the three HMA ^15^N labels, we constructed a four‐spin model (Figure S3b,c). Each nitrogen atom is 1.3 Å from the guanidinium ^13^C, and the N—C—N bond angles are 120°. Given this symmetry, we used the distance from the guanidinium ^13^C to the protein ^13^C to parameterize the three ^13^C–^15^N distances. We consider two orientations of approach of a protein ^13^C to the guanidinium group. In the first orientation, the ^13^C–^13^C vector is coplanar with the three ^15^N spins and also colinear with one of the three C—N bonds. The HMA ^15^N atom between the two carbons has the shortest distance ^13^C–^15^N distance among the three distances. In the second orientation, the ^13^C–^13^C vector is perpendicular to the plane of the three nitrogen atoms, thus the three ^13^C–^15^N distances are similar. For each observed REDOR dephasing curve, the lateral approach gives the longest ^13^C–^13^C distance (*d*_max_) whereas the vertical approach gives the shortest ^13^C–^13^C distances (*d*_min_). Based on these two models, we simulated the ^13^C–^15^N REDOR curves to obtain the distance range, denoted by *d*_min_ and *d*_max_. These REDOR simulations include all three ^13^C–^15^N dipolar couplings and the three ^15^N–^15^N couplings. The main CSA principal axis of ^15^N in the NH group is approximated as along the N—H bond (Figure S3b,c). For the two NH_2_ groups, the main ^15^N CSA principal axis is assumed to bisect the H—N—H bond angle. The ^15^N principal values are taken from the literature (Duncan, 1997).

^1^H–^19^F REDOR simulations were conducted using a three‐spins system (Figure S3d). The F—C—F bond angle is 108° and the C—F bond length is 1.3 Å. Although the ring carbon bonded to the two fluorines is not labeled, we use its distance to the protein H^N^ (*d*_CH_) to parameterize the two H^N^–F distances. We use a geometry in which the C–H^N^ vector bisects the F—C—F angle, and the two ^19^F atoms point away from the H^N^. This geometry gives the minimum distance. In the simulation, both the ^19^F–^19^F dipolar coupling and the two ^1^H–^19^F dipolar couplings are included. The main ^19^F CSA principal axis is chosen to lie along the C—F bond, and literature values for the principal values of a CF_2_ group are used (Duncan, 1997).

All REDOR simulations were carried out in 0.1 Å increments between 2 and 20 Å. For each distance, the simulations consider the background channel pulses to have flip angles of 145° to 180° in 5° increments. This background channel is ^19^F for ^13^C–^19^F and ^1^H–^19^F REDOR, and ^15^N for ^13^C–^15^N REDOR. The pulse flip angle distribution accounts for rf field inhomogeneity and the resulting pulse imperfection. The different flip angles were weighted by a half‐Gaussian with mean 180° and standard deviation 15°. The simulated REDOR *S*/*S*_0_ values were scaled to the correct stoichiometry and compared to the measured *S*/*S*_0_ values.

### 4.9 Distance‐restrained docking of HMA to ETM

Seven best‐fit REDOR distances (Table S3) were used to dock HMA into the low‐pH structure of ETM (Medeiros‐Silva et al., 2023). Docking was conducted using the HADDOCK 2.4 web interface (de Vries et al., 2010; van Zundert et al., 2016). In one set of docking simulations, the measured REDOR distances were inputted as unambiguous interaction restraints, which are always enforced (Table S4). Additional restraints were provided to restrain the monomers of the helical bundle together during the refinement stage of HADDOCK. These restraints consist of two restraints between each pair of monomers within the pentamer, thus there are 20 of these restraints total. They are generated using the restrain‐bodies script in the HADDOCK‐tools Python utilities, which measures them on the apo ETM structure. These same restraints were used in all drug docking in this work. The N‐ and C‐terminal residues of ETM (E8 and R38) were specified to be charged. Based on the observed correlation peaks and REDOR dephasing, the list of “active” residues was set to be V14, L19, F20, A22, F23, V24, F26, T30, A32, I33, T35, A36. These active residues were set on two monomers, and the entire HMA molecule was marked as active. The solvent was DMSO, and all other parameters were the default values of the software for ligand docking.

To determine if any measured contacts represent alternative binding modes, we also conducted a second set of docking in which the seven REDOR distances were provided as ambiguous interaction restraints (Table S4). In this mode, no active residue list is provided. The only unambiguous restraints provided were the abovementioned 20 restraints that maintain the pentamer topology of ETM.

In either docking procedure, three runs were conducted to assess how the drug interacts with multiple subunits of the ETM pentamer. The ^13^C–^15^N REDOR data restrained the HMA guanidinium group, whereas the ^1^H–^19^F REDOR data restrained the hexamethylene ring. We assume that each end of the HMA molecule contacts a single monomer of the helical bundle. Thus, we performed three HADDOCK simulations where the two types of restraints were assigned to one ETM monomer, or two adjacent monomers *i* and *i* + 1, or two adjacent monomers in the opposite order, *i* and *i* − 1. In each docking run, the top five lowest scoring clusters were chosen to evaluate the binding of HMA in ETM.

## AUTHOR CONTRIBUTIONS

**Noah H. Somberg**: Data curation, formal analysis, investigation, writing‐original draft, writing‐review and editing. **João Medeiros‐Silva**: Data curation, formal analysis, editing. **Hyunil Jo**: Data curation. **Jun Wang**: Data curation. **William F. DeGrado**: Resources, writing‐review and editing. **Mei Hong**: Conceptualization, formal analysis, resources, supervision, writing—review and editing.

## CONFLICT OF INTEREST STATEMENT

The authors declare no potential conflict of interest.

## Supporting information


**Data S1.** Supporting Information

**Table S1.** NMR samples used to investigate HMA binding to SARS‐CoV‐2 ETM.
**Table S2.** NMR experimental conditions.
**Table S3.** Measured HMA‐ETM distances.
**Table S4.** Distance‐restrained docking with HADDOCK and Δ*G* prediction.
