# Supplementary material for: Hexamethylene amiloride binds the SARS‐CoV‐2 envelope protein at the protein–lipid interface
Source: Protein Sci. 2023 Oct 1;32(10):e4755. doi: 10.1002/pro.4755 (PMC10503410; doi:10.1002/pro.4755)
Supplement: Supplementary file 1 — Data S1. Supporting Information [file PRO-32-e4755-s002.docx]

**Supporting Information**

**Hexamethylene Amiloride Binds the SARS-CoV-2 Envelope Protein at the Protein-Lipid Interface**

Noah H Somberg,^1^ João Medeiros-Silva,^1^ Hyunil Jo^2^, Jun Wang^3^, William F DeGrado^2^ and Mei Hong^1*^

^1^Department of Chemistry, Massachusetts Institute of Technology, 170 Albany Street, Cambridge, MA 02139

^2^Department of Pharmaceutical Chemistry, University of California San Francisco, 555 Mission Bay Blvd. South, San Francisco, CA 94158

^3^Department of Medicinal Chemistry, Ernest Mario School of Pharmacy, Rutgers, the State University of New Jersey, Piscataway, NJ 08854

* Corresponding author: Professor Mei Hong, meihong@mit.edu


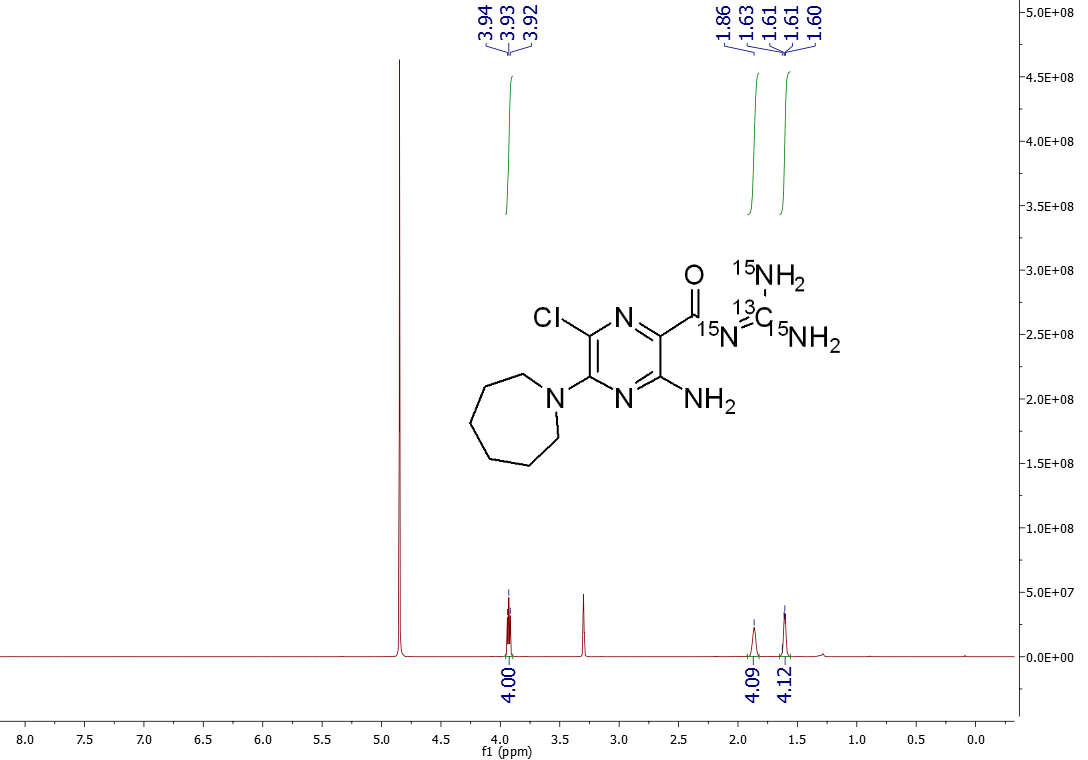


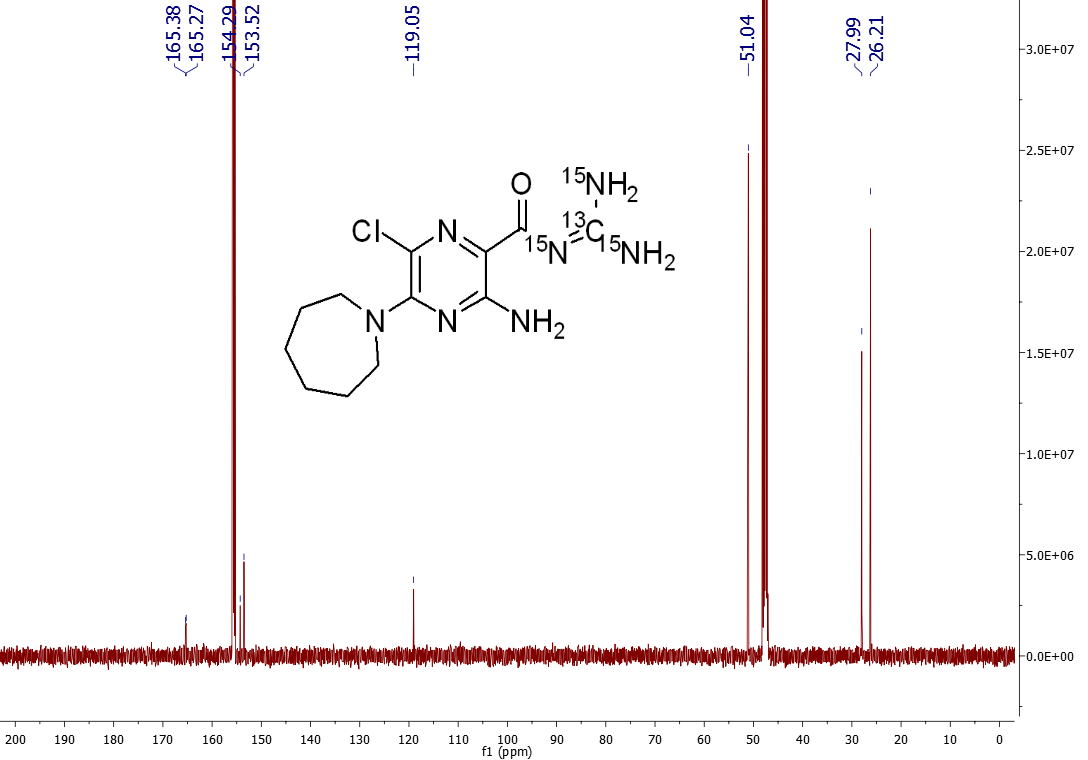


**Figure S1**. ^1^H (top) and ^13^C (bottom) solution NMR spectra of ^13^C, ^15^N-labeled HMA.


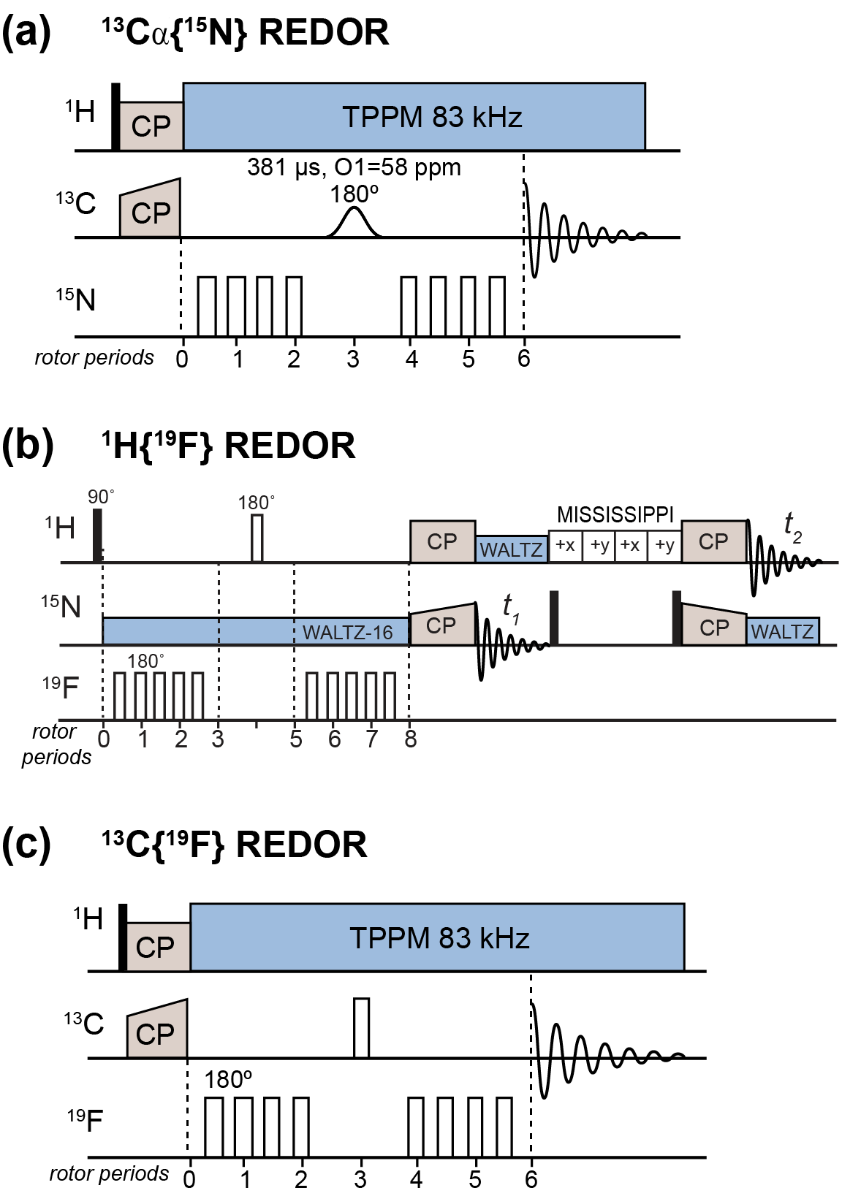


**Figure S2**. Pulse sequence diagrams for the REDOR experiments used in this work. (**a**) Frequency-selective ^13^Cα-^15^N REDOR experiment ^36^. (**b**) ^1^H-detected ^1^H-^19^F REDOR experiment ^38^. (**c**) Broadband ^13^C-^19^F REDOR experiment.


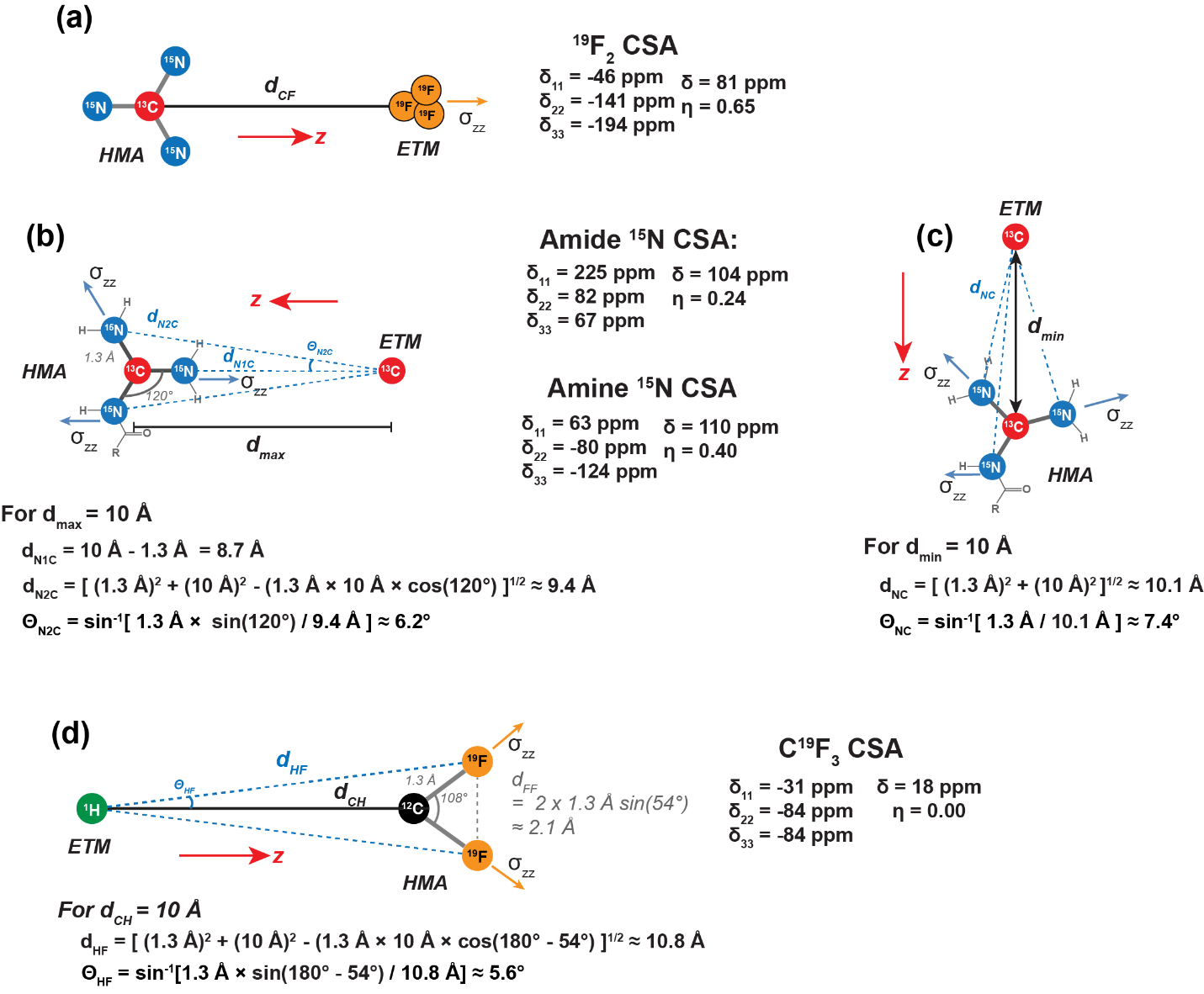


**Figure S3**. Geometric models, spin systems, and chemical shift tensors used in the REDOR simulations in this work. (**a**) ^13^C-^19^F REDOR between ^13^C-labeled HMA and CF_3_-labeled ETM. The distance d_CF_ is from the guanidinium ^13^C to the center of gravity of the three methyl fluorines. (**b, c**) ^13^C-^15^N REDOR of ^13^C-labeled ETM and ^15^N-labeled HMA guanidinium. (**b**) When ETM ^13^C is parallel to an HMA ^13^C-^15^N bond, the distance d_max_ is the upper-bound value for the measured REDOR dephasing. (**c**) When ETM ^13^C is perpendicular to the plane of the three nitrogens, the distance d_min_ is the lower-bound value for the measured dephasing. (**d**) ^1^H-^19^F REDOR of H^N^-labeled ETM and F_2_-HMA. The two H-F distances are parameterized by d_CH_. ^19^F and ^15^N chemical shift tensor values shown here are approximate values and are obtained from the literature ^44^ for the closest chemical structures. This chemical shift tensor approximation does not affect the distance quantification within the experimental uncertainty.


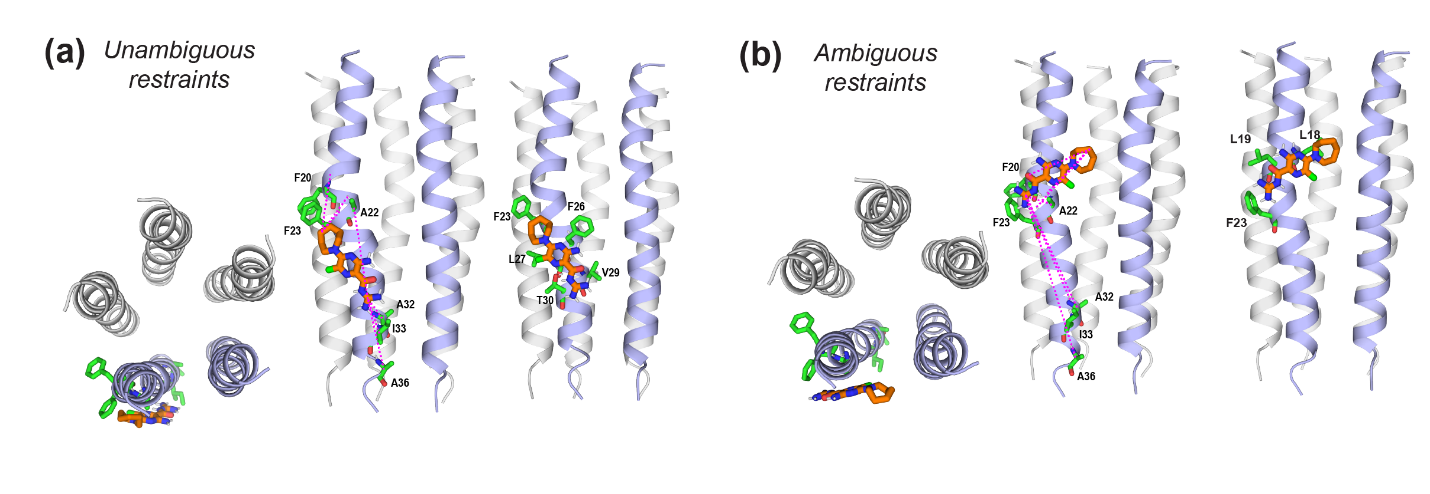


**Figure S4.** Distance restrained docking of HMA (orange) to the ETM pentamer at acidic pH, interacting with a single helix. (**a**) Lowest energy pose from single helix docking with unambiguous restraints. Similar interactions are observed as in the “bridging” pose (Fig. 8a). (b) Lowest energy pose from ambiguous restraints to a single helix. A similar pose is observed in the “tangential” pose, see Fig. 8c.

**Example SIMPSON input file, for ^13^C-^15^N REDOR, d_max_ = 10 Å, 175° ^15^N pulses**

spinsys {

channels 13C 15N

nuclei 13C 15N 15N 15N

dipole 1 2 {[dist2dip 13C 15N 8.70]} 0 0.00 0

dipole 1 3 {[dist2dip 13C 15N 10.40]} 0 6.21 0

dipole 1 4 {[dist2dip 13C 15N 10.40]} 0 -6.21 0

dipole 2 3 {[dist2dip 15N 15N 2.06]} 0 30.00 0

dipole 3 4 {[dist2dip 15N 15N 2.06]} 0 -30.00 0

dipole 2 4 {[dist2dip 15N 15N 2.06]} 0 90.00 0

shift 1 0p 0p 0 50 20 10

shift 2 0p 110p 0.40 0 180 0

shift 3 0p 110p 0.40 0 60 0

shift 4 0p 104p 0.24 0 0 0

}

par {

variable index 1

np 512

spin_rate 10500

proton_frequency 800e6

start_operator I1x

detect_operator I1p

method direct

crystal_file rep168

gamma_angles 64

sw spin_rate/2

variable tsw 1e6/sw

verbose 1101

variable rfF1 62500

variable rfF2 35714 * 175 / 180.0

variable rfF3 35714

variable t180F1 0.5e6/rfF1

variable t180F2 0.5e6/rfF3

variable tr1 0.5e6/spin_rate-0.5*t180F1-0.5*t180F2

variable tr2 0.5e6/spin_rate-t180F2

}

proc pulseq {} {global par

maxdt 0.5

reset

delay $par(tr2)

pulse $par(t180F2) 0 x $par(rfF2) x

delay $par(tr2)

pulse $par(t180F2) 0 x $par(rfF2) y

store 1

reset

acq

delay $par(tr2)

pulse $par(t180F2) 0 x $par(rfF2) x

delay $par(tr1)

pulse $par(t180F1) $par(rfF1) x 0 x

delay $par(tr1)

pulse $par(t180F2) 0 x $par(rfF2) x

delay $par(tr2)

pulse $par(t180F2) 0 x $par(rfF2) y

store 2

acq

for {set i 2} {$i < $par(np)} {incr i} {

reset

prop 1

prop 2

prop 1

store 2

acq

}

}

proc main {} {

global par

set f [fsimpson]

fsave $f $par(name).crv

}
