# Supplementary material for: Hexamethylene amiloride binds the SARS‐CoV‐2 envelope protein at the protein–lipid interface
Source: Protein Sci. 2023 Oct 1;32(10):e4755. doi: 10.1002/pro.4755 (PMC10503410; doi:10.1002/pro.4755)
Supplement: Supplementary file 2 — Table S1. NMR samples used to investigate HMA binding to SARS‐CoV‐2 ETM. Table S2. NMR experimental conditions. Table S3. Measured HMA‐ETM distances. Table S4. Distance‐restrained docking with HADDOCK and ΔG prediction. [file PRO-32-e4755-s001.pdf]

Table S1. NMR samples used to investigate HMA binding to SARS-CoV-2 ETM.

Sample ID #	Description	Protein and initial solvent	Drug and initial solvent	Membrane and initial solvent	P : D : L molar ratio	MAS rotor	Buffer	Figures
1	HMA only, DMPX, pH 7.5	-	CN-HMA (1 mg) TFE	DMPC:PG 8:2 (12 mg) Chloroform	0 : 20 : 100	3.2 mm	pH 7.5 (25 mM Tris, 1 mM EDTA, 0.07 NaN3, 25 mM NaCl)	2a, 2b (top)
2	ETM only, DMPX, pH 7.5	CN-ETM (2 mg) TFE	-	DMPC:PG 8:2 (8 mg) Chloroform	5 : 0 : 100	3.2 mm	pH 7.5 (25 mM Tris, 1 mM EDTA, 0.07 NaN3, 25 mM NaCl)	2a, 2b (middle)
3	HMA, ETM, DMPX, pH 7.5	CN-ETM (3 mg) TFE	CN-HMA (1 mg) TFE	DMPC:PG 8:2 (12 mg) Chloroform	5 : 20 : 100	3.2 mm	pH 7.5 (25 mM Tris, 1 mM EDTA, 0.07 NaN3, 25 mM NaCl)	2a, 2b (bottom)
4	HMA, ETM, DMPX, pH 4.5	C-ETM (5 mg) Methanol	CN-HMA (0.4 mg) TFE	DMPC:PG 7:3 (15 mg) Chloroform	5 : 5 : 75	3.2 mm	pH 4.5 (25 mM Acetate, 0.07 NaN3, 25 mM NaCl)	2c, 2d, 6b, 6c, 6d
5	F2HMA only, DMPX, pH 7.5	-	F2-HMA (0.3 mg), DMSO	DMPC:PG 7:3 (6 mg) Chloroform	0 : 5 : 50	1.9 mm	pH 7.5 (25 mM Tris, 1 mM EDTA, 0.07 NaN3, 25 mM NaCl)	3a
6	F2HMA, V14F-ETM, DMPX, pH 4.5	V14F-ETM (3 mg) Methanol	F2-HMA (0.3 mg) DMSO	DMPC:PG 7:3 (6 mg) Chloroform	5 : 5 : 50	1.9 mm	pH 4.5 (25 mM Acetate, 0.07 NaN3, 25 mM NaCl)	3b, 5a
7	F2HMA, V14F-ETM, DMPX, pH 7.5	V14F-ETM (2 mg) Methanol	F2-HMA (0.1 mg) TFE	DMPC:PG 7:3 (6 mg) Chloroform	5 : 2 : 50	1.9 mm	pH 7.5 (25 mM Tris, 1 mM EDTA, 0.07 NaN3, 25 mM NaCl)	3c
8	HMA, V14F-ETM, DMPX, pH 7.5	V14F-ETM (3 mg) TFE	CN-HMA (0.1 mg) TFE	DMPC:PG 7:3 (6 mg) Chloroform	5 : 2 : 50	1.9 mm	pH 7.5 (25 mM Tris, 1 mM EDTA, 0.07 NaN3, 25 mM NaCl)	5b (left)
9	HMA, V14F-ETM, DMPX, pH 4.5	V14F-ETM (3 mg) Methanol	CN-HMA (0.3 mg) TFE	DMPC:PG 7:3 (6 mg) Chloroform	5 : 5 : 50	1.9 mm	pH 4.5 (25 mM Acetate, 0.07 NaN3, 25 mM NaCl)	5b (right)
10	F2HMA, ETM, pH 4.5	CDN-ETM (3 mg) Methanol	F2-HMA (0.3 mg) DMSO	d54-DMPC:PG 7:3 (6 mg) Chloroform	5 : 5 : 50	1.9 mm	pH 4.5 (25 mM Acetate, 0.07 NaN3, 25 mM NaCl)	7a

Table S2. NMR experimental conditions

Sample ID #	Description	Figure Ref	NMR experiment	NMR conditions and parameters	Acquisition
6	F2HMA, V14F-ETM, DMPX, pH 4.5	4a	2D FF CORD	B_0 = 14.1 T; T = 272 K; ν_{MAS} = 25 kHz; $\nu_{1H(exc)}$ = 71 kHz; $\nu_{19F(exc)}$ = 71 kHz; $\nu_{1H(HF)}$ = 90 kHz; $\nu_{19F(HF)}$ = 65 kHz; τ_{HC} = 0.5 ms; τ_{CORD} = 300 ms; TPPM decoupl. 71 kHz;	ns = 616; τ_{rd} = 1.5 s; τ_1= 1.2 ms; τ_{acq} = 3.6 ms; Expt = 36.5 h
8	HMA, V14F-ETM, DMPX, pH 7.5	4b (left)	1D C-F REDOR	B_0 = 14.1 T; T = 263 K; ν_{MAS} = 10.5 kHz; $\nu_{1H(exc)}$ = 71 kHz; $\nu_{13C(exc)}$ = 62 kHz; $\nu_{1H(HC)}$ = 60 kHz; $\nu_{13C(HC)}$ = 50 kHz; τ_{HC} = 1.5 ms; $\nu_{19F(REDOR)}$ = 71 kHz; τ_{REDOR} = [8.0, 10.6, 12.0, 12.2] ms; $N_{tr,REDOR}$ = [64, 112, 126, 128], TPPM decoupl. 92 kHz;	ns (S and S0 each) = [16384, 36864, 8192, 32768]; τ_{rd} = 1.6 s; τ_{acq} = 12.8 ms; Expt = [7.2 x 2 = 14.6, 16.4 x 2 = 32.8, 3.6 x 2 = 7.2, 14.6 x 2 = 29.2] h; Total expt = 83.8 h
9	HMA, V14F-ETM, DMPX, pH 4.5	4b (right)	1D C-F REDOR	B_0 = 14.1 T; T = 263 K; ν_{MAS} = 10.5 kHz; $\nu_{1H(exc)}$ = 71 kHz; $\nu_{13C(exc)}$ = 62 kHz; $\nu_{1H(HC)}$ = 60 kHz; $\nu_{13C(HC)}$ = 50 kHz; τ_{HC} = 1.8 ms; $\nu_{19F(REDOR)}$ = 71 kHz; τ_{REDOR} = [5.1, 8.0, 10.6, 12.0] ms; $N_{tr,REDOR}$ = [54, 84, 112, 126], TPPM decoupl. 71 kHz;	ns (S and S0 each) = [24576, 24576, 40960, 40960]; τ_{rd} = 1.6 s; τ_{acq} = 12.8 ms; Expt = [12.3 x 2 = 24.6, 12.3 x 2 = 24.6, 20.5 x 2 = 41.0, 20.5 x 2 = 41.0] h; Total expt = 131.2 h
4	HMA, ETM, DMPX, pH 4.5	5b	1D NHHC	B_0 = 21.1 T; T = 276 K; ν_{MAS} = 11.8 kHz; $\nu_{1H(exc)}$ = 83 kHz; $\nu_{13C(exc)}$ = 62 kHz; $\nu_{1H(HN)}$ = 44 kHz; $\nu_{15N(HN)}$ = 32 kHz; $\nu_{1H(HC)}$ = 62 kHz; $\nu_{13C(HC)}$ = 50 kHz; τ_{HN} = 1.0 ms; τ_{NH} = 1.0 ms; τ_{HC} = 0.8 ms; $\tau_{spin\text{-}diffusion}$ = [0.5, 1.0] ms; TPPM decoupl. 83 kHz;	ns = [45056, 122880]; τ_{rd} = 1.7 s; τ_{acq} = 15.4 ms; Expt = [21.3, 58.0] h; Total expt = 79.3 h
		5d	2D CC CORD	B_0 = 18.8 T; T = 274 K; ν_{MAS} = 10.5 kHz; $\nu_{1H(exc)}$ = 83 kHz; $\nu_{13C(exc)}$ = 62 kHz; $\nu_{1H(HC)}$ = 61 kHz; $\nu_{13C(HC)}$ = 50 kHz; τ_{HC} = 0.5 ms; τ_{CORD} = 23 ms; TPPM decoupl. 83 kHz;	ns = 36; τ_{rd} = 1.7 s; τ_1= 1.5 ms; τ_{acq} = 10.0 ms; Expt = 13.6 h
		5c,f	1D selective C-N REDOR	B_0 = 18.8 T; T = 274 K; ν_{MAS} = 10.5 kHz; $\nu_{1H(exc)}$ = 83 kHz; $\nu_{13C(exc)}$ = 62 kHz; $\nu_{1H(HC)}$ = 61 kHz; $\nu_{13C(HC)}$ = 50 kHz; τ_{HC} = 0.5 ms; Offset13C = 58.0 ppm; $\tau_{selective\ 13C,gaussian}$ = 0.385 ms; $N_{tr,selective\ 13C}$ = 4; $\nu_{15N(REDOR)}$ = 36 kHz; τ_{REDOR} = [12.0, 20.0] ms; $N_{tr,REDOR}$ = [126, 210]; TPPM decoupl. 83 kHz;	ns (S and S0 each) = [16384, 32768]; τ_{rd} = 2.0 s; τ_{acq} = 15.4 ms; Expt = [9.1 x 2 = 18.2, 18.2 x 2 = 36.4] h; Total expt = 54.6 h
10	F2HMA, ETM, pH 4.5	6a,c,d	2D hNH H-F REDOR	B_0 = 14.1 T; T = 268 K; ν_{MAS} = 38 kHz; $\nu_{1H(exc)}$ = 71 kHz; $\nu_{13C(exc)}$ = 62 kHz; $\nu_{1H(HC)}$ = 88 kHz; $\nu_{15N(HN)}$ = 50 kHz; τ_{HN} = 1.5 ms; τ_{HN} = 0.4 ms; τ_{solsup} = 0.25 ms; $\nu_{1H(solsup)}$ = 7 kHz; $\nu_{15N(dec,REDOR,aq)}$ = 7 kHz; $\nu_{19F(REDOR)}$ = 83 kHz; τ_{REDOR} = [2.53, 4.53, 7.00, 10.00] ms; $N_{tr,REDOR}$ = [96, 172, 266, 380]	ns (S and S0 each) = [64, 80, 112, 80]; τ_{rd} = 1.4 s; τ_1= 27 ms; τ_{acq} = 15.4 ms; Expt = [4.5 x 2 = 9.0, 5.6 x 2 = 11.2, 7.8 x 2 = 15.7, 5.6 x 2 = 11.2] h; Total expt = 47.1 h

Table S3. Measured HMA-ETM distances

Protein atoms	HMA atoms	Parameter Distance	best-fit distances, 20% (χ^2_ν)			Experiment	Distance used in docking	CNS Format Restraint fed to HADDOCK
			dmin (20%)	dmax (20%)	full distance (20%)			
I33 Cα	HMA N_3	I33 Cα - HMA Cζ	5.1 ± 0.1 Å (0.50)	5.8 ± 0.1 Å (0.51)	5.5 ± 0.5 Å	C{N} REDOR	Y	assign (resid 33 and name CA) (resid 0 and name C1) 5.5 2.5 1.4
L? Cα	HMA N_3	L? Cα - HMA Cζ	5.8 ± 0.1 Å (3.75)	6.4 ± 0.2 Å (3.85)	6.1 ± 0.5 Å	C{N} REDOR	N	
A22 Cα	HMA N_3	A22 Cα - HMA Cζ	5.9 ± 0.2 Å (0.11)	6.5 ± 0.1 Å (0.04)	6.1 ± 0.4 Å	C{N} REDOR	Y	assign (resid 22 and name CA) (resid 0 and name C1) 6.2 3.2 1.5
A32 Cα	HMA N_3	A32 Cα - HMA Cζ	5.4 ± 0.1 Å (0.05)	6.1 ± 0.1 Å (0.11)	5.3 ± 0.5 Å	C{N} REDOR	Y	assign (resid 32 and name CA) (resid 0 and name C1) 5.7 2.7 1.4
A36 Cα	HMA N_3	A36 Cα - HMA Cζ	5.1 ± 0.1 Å (2.58)	5.9 ± 0.1 Å (2.60)	5.0 ± 0.5 Å	C{N} REDOR	Y	assign (resid 36 and name CA) (resid 0 and name C1) 5.5 2.5 1.4
A22 H^N	HMA F_2	A36 HN - HMA C9			8.9 ± 0.7 Å (0.78)	H{F} REDOR	Y	assign (resid 422 and name HN) (resid 0 and name C9) 9.0 6.0 1.6
F20/F23 H^N	HMA F_2	A36 HN - HMA C9			8.6 ± 0.8 Å (0.82)	H{F} REDOR	Y	assign (resid 420 and name HN) (resid 0 and name C9) 8.7 5.7 1.7
								assign (resid 423 and name HN) (resid 0 and name C9) 8.7 5.7 1.7
V14F-CF_3	HMA Cζ	V14F-CF_3 - HMA Cζ			10.6 ± 1.1 Å (0.72)	C{F} REDOR	N	
V14F-CF_3	HMA F_2	V14F-CF_3 - HMA F_2			< 20 Å	FF spin diffusion	N	

Table S4. Distance-restratined docking with HADDOCK and ΔG prediction

Pose Name	Figure	Restraint Type	HADDOCK score	Rank of Cluster	Cluster Size	$\Delta G_{predicted}$ (kcal/mol)	ΔG score	Kd (μM)	Restraint Directionality
Bridging	7a	Unambiguous	0.332 ± 1.617	#1	135	-5.98	166.43	41.1	HMA-guanidinium to monomer 1, HMA-hexamethylene to monomer 5
Parallel	7b	Unambiguous	1.597 ± 1.266	#2	6	-6.16	163.13	30.3	HMA-guanidinium to monomer 1, HMA-hexamethylene to monomer 5
Tangential	7c	Ambiguous	-29.108 ± 0.202	#1	48	-6.16	160.96	30.3	HMA-guanidinium to monomer 1, HMA-hexamethylene to monomer 5
Intercalating	7d	Ambiguous	-30.229 ± 2.258	#1	10	-5.92	168.44	45.5	HMA-guanidinium to monomer 5, HMA-hexamethylene to monomer 1
